# Expanding the Chemical Space of Tetracyanobuta‐1,3‐diene (TCBD) through a Cyano‐Diels‐Alder Reaction: Synthesis, Structure, and Physicochemical Properties of an Anthryl‐fused‐TCBD Derivative

**DOI:** 10.1002/chem.202103079

**Published:** 2021-10-12

**Authors:** Luis M. Mateo, Luca Sagresti, Yusen Luo, Dirk M. Guldi, Tomas Torres, Giuseppe Brancato, Giovanni Bottari

**Affiliations:** ^1^ Departamento de Química Orgánica Universidad Autónoma de Madrid Campus de Cantoblanco 28049 Madrid Spain; ^2^ IMDEA-Nanociencia Faraday 9, Campus de Cantoblanco 28049 Madrid Spain; ^3^ Institute for Advanced Research in Chemical Sciences (IAdChem) Universidad Autónoma de Madrid 28049 Madrid Spain; ^4^ Scuola Normale Superiore and CSGI Piazza dei Cavalieri 7 56126 Pisa Italy; ^5^ Istituto Nazionale di Fisica Nucleare Largo Pontecorvo 3 56100 Pisa Italy; ^6^ Department of Chemistry and Pharmacy, Interdisciplinary Center for Molecular Materials (ICMM) Friedrich-Alexander-Universität Erlangen-Nürnberg Egerlandstr. 3 91058 Erlangen Germany

**Keywords:** anthryl-fused derivative, cyano-Diels-Alder reaction, electron acceptor, photophysics, tetracyanobuta-1,3-diene

## Abstract

Tetracyanobuta‐1,3‐diene (TCBD) is a powerful and versatile electron‐acceptor moiety widely used for the preparation of electroactive conjugates. While many reports addressing its electron‐accepting capability have appeared in the literature, significantly scarcer are those dealing with its chemical modification, a relevant topic which allows to broaden the chemical space of this interesting functional unit. Here, we report on the first example of a high‐yielding cyano‐Diels‐Alder (CDA) reaction between TCBD, that is, where a nitrile group acts as a dienophile, and an anthryl moiety, that is, acting as a diene. The resulting anthryl‐fused‐TCBD derivative, which structure was unambiguously identified by X‐ray diffraction, shows high thermal stability, remarkable electron‐accepting capability, and interesting electronic ground‐ and excited‐state features, as characterized by a thorough theoretical, electrochemical, and photophysical investigation. Moreover, a detailed kinetic analysis of the intramolecular CDA reaction transforming the anthryl‐TCBD‐based reactant into the anthryl‐fused‐TCBD product was carried out at different temperatures.

## Graphical abstract

**Tetracyanobuta‐1,3‐diene** (TCBD) is a powerful and versatile electron‐acceptor moiety widely used for the preparation of electroactive conjugates. Here, we expand the chemical space of such moiety through a high‐yielding cyano‐Diels‐Alder synthesis of a novel, thermally‐stable anthryl‐fused‐TCBD derivative which presents intriguing physicochemical properties.

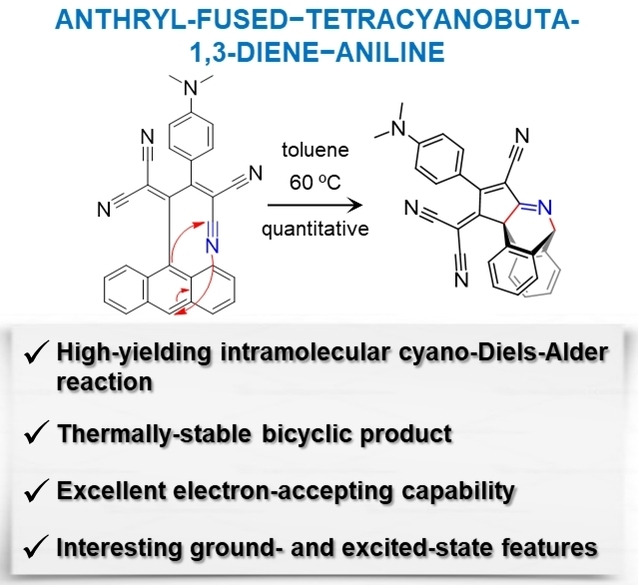

## Introduction

The preparation of novel electron donor‐acceptor (D−A) systems and studies thereof represent a transdisciplinary research area. It directly impacts technologically relevant fields such as light‐to‐energy conversion schemes and organic electronics.[^1^, ^2^, ^3^] While the portfolio of electron donors is large, versatile, and continuously growing, the one of electron acceptors is mostly centered around fullerenes, perylene diimides, and multicyano fragments, which are among the most investigated building blocks.

Within the class of multicyano fragments, tetracyanobuta‐1,3‐dienes (TCBDs) are at the center of attention, especially in push‐pull conjugates.^[4]^ *Per se*, TCBDs are powerful electron acceptors, which are easily integrated on activated alkynes through a [2+2] cycloaddition‐retroelectrocyclization (CA‐RE) reaction with tetracyanoethylene (TCE).^[5]^ As a consequence, TCBDs have been combined with, for example, triphenylamine,[^6^, ^7^, ^8^] BODIPY,[^9^, ^10^] truxenes,^[11]^ fullerenes,^[12]^ corannulenes,^[13]^ (sub)porphyrins,[^14^, ^15^, ^16^, ^17^] and (sub)phthalocyanines[^18^, ^19^, ^20^, ^21^, ^22^] towards the realization of D−A conjugates. Next to being an outstanding electron acceptor, the TCBD structure is peculiar owing to the quasi‐orthogonal arrangement of its two dicyanovinyl (DCV) halves^[5]^ and the restricted rotation around the C−C bond connecting them. As a result, atropisomers can be formed, which under some experimental conditions have even been isolated.[^12^, ^19^]

Furthermore, in a few interesting cases, chemical transformations of TCBD into highly‐functionalized multicyano derivatives through intra‐[^23^, ^24^, ^25^] or intermolecular[^26^, ^27^] reactions have also been reported.

Aza‐Diels‐Alder (ADA) reactions are [4+2] cycloadditions between a diene and a dienophile containing a nitrogen atom either on one or both reactants, leading to unsaturated six‐membered *N*‐heterocycles. ADA reactions have been presented in numerous reports,[^28^, ^29^] whereas scarcer are those focusing on cyano‐Diels‐Alder (CDA) reactions, a type of ADA reaction in which a cyano moiety is the dienophile.[^30^, ^31^, ^32^] Both the high thermal stability and dissociation energy of the C−N triple bond render the nitrile a poor dienophile for cycloadditions. To overcome these limitations, several strategies have been developed. For example, increasing the nitrile dienophilic character by incorporating electron‐withdrawing groups next to it,[^33^, ^34^, ^35^] or by promoting entropically‐favourable intramolecular reactions.^[36]^ Yet, high temperatures are usually required to activate CDA reactions, though this also favours the retro‐CDA process partially leading to the starting materials. This calls for shifting the reaction equilibrium towards the products by stabilizing CDA adducts through the complexation with external species.^[30]^

As part of our interest to diversify the TCBD toolbox, we decided to expand the chemical space of this electroactive moiety by exploring its possible application in CDA reactions. We reasoned that TCBD with its multiple, closely‐spaced electron‐withdrawing cyano moieties constitutes a resourceful platform for intramolecular CDA reactions if integrated within a suitable molecular framework. Indeed, the resulting multicyano CDA products could present appealing physicochemical features, different to those of their TCBD precursors.

Here, we report on the high‐yielding synthesis of a novel, thermally‐stable anthryl‐fused‐TCBD derivative. Its synthesis is based on an intramolecular CDA reaction between TCBD, that is, where a nitrile group acts as a dienophile, and an anthryl moiety, that is, acting as a diene. A detailed kinetic analysis carried out at different temperatures showed that such an intramolecular CDA reaction proceeds as a reversible reaction, though strongly shifted towards the cycloadduct. Such a CDA product, whose bicyclic structure was unambiguously corroborated by X‐ray diffraction analysis, was thoroughly characterized by theoretical, electrochemical, and photophysical investigation. Results showed that the anthryl‐fused‐TCBD derivative presents interesting ground‐ and excited‐state features, and remarkable electron‐accepting capability.

## Results and Discussion

**Scheme 1:**
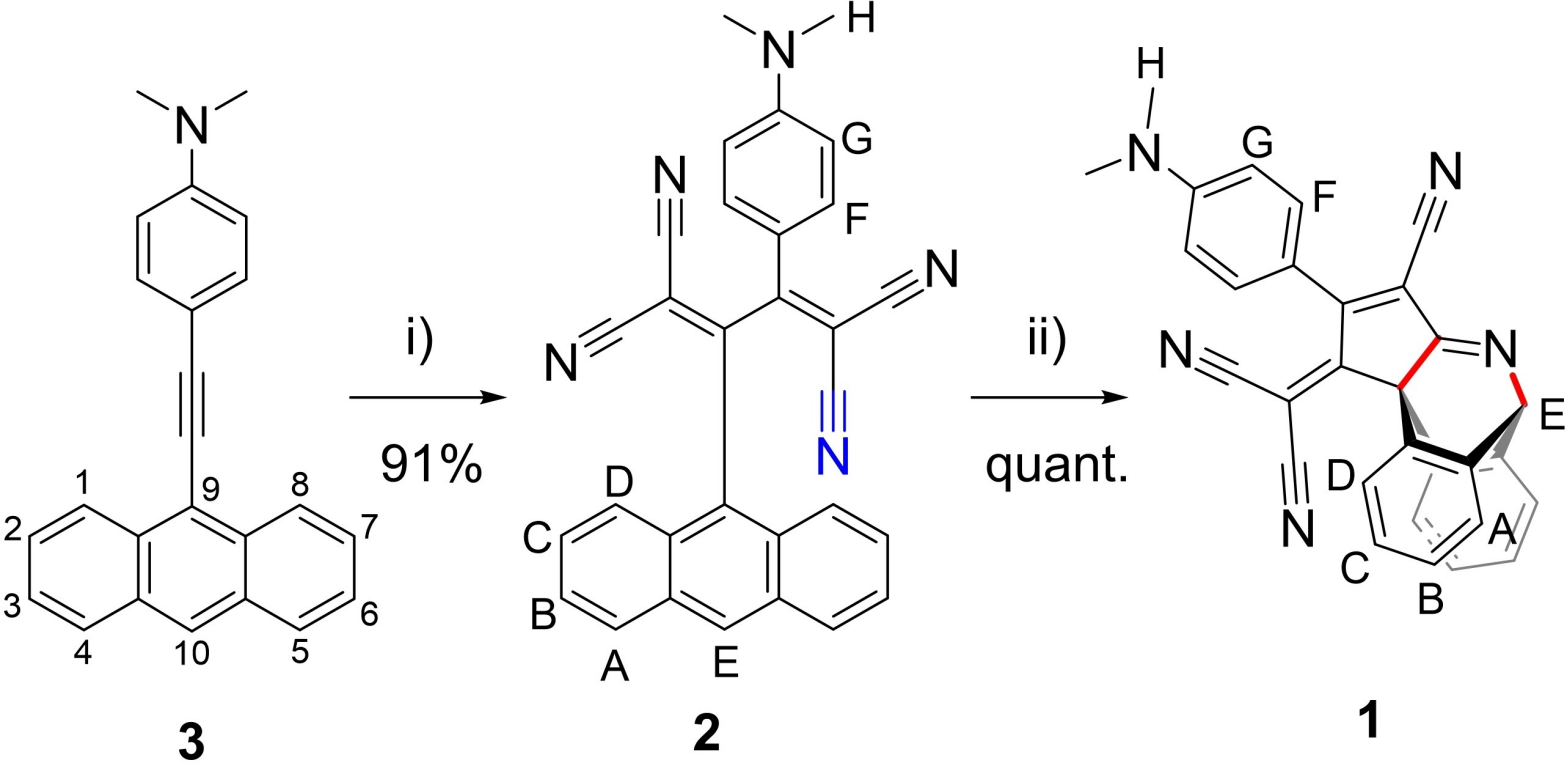
Synthetic route to anthryl‐fused‐TCBD‐aniline **1**. i) Tetracyanoethylene, THF, 40 °C, 16 h; ii) toluene, 70 °C, 12 h. The numbering of the anthryl moiety positions is presented for derivative **3**.

Anthryl‐fused‐TCBD‐aniline derivative **1** was prepared in a two‐step synthesis starting from 4‐(anthracen‐9‐ylethynyl)‐*N*,*N*‐dimethylaniline **3**,^[37]^ which was reacted with TCE affording TCBD‐functionalized compound **2** in an excellent yield (Scheme 1).^[4]^ Heating a toluene solution of **2** at 70 °C promoted its quantitative transformation into anthryl‐fused‐TCBD‐aniline **1** through an intramolecular CDA reaction (Scheme 1). **1** was stable over several weeks in solution, a stability not observed for a previously reported CDA derivative, which could be obtained in good yield only if “stabilized” by N→B coordination of its imine nitrogen with B(C_5_F_5_)_3_, a strong Lewis acid.^[30]^ On the contrary, in our case, addition of the latter borane had an effect exclusively on the **2→1** CDA reaction rate, which increased dramatically (see below). We reckon that the presence of multiple cyano groups are crucial for the high yielding intramolecular CDA reaction transforming **2** into to **1** as well as for the high thermodynamic stability of the latter product (see below). Reasonably, these electron‐withdrawing moieties help i) to increase the dienophile character of the cyano group involved in the intramolecular CDA (colored in blue in Scheme 1), and ii) to stabilize the resulting CDA product in which two new C−C and C−N single bonds are formed (colored in red in Scheme 1) (see below). It is also important to point out that the introduction in **2** of the TCBD at the 9‐position of an anthryl moiety is crucial to promote the intramolecular CDA reaction. In this context, when the TCBD unit was introduced at the 2‐position of an anthryl group, no intramolecular CDA reaction took place.^[38]^

Derivative **1**, as well as its anthryl‐based precursors **2** and **3**, were fully characterized by a wide range of spectroscopic, spectrometric, and electrochemical techniques (see Supporting Information).

**Figure 1:**
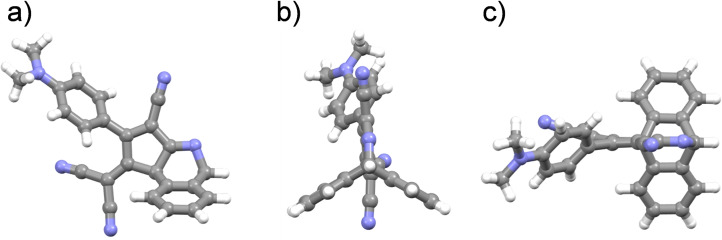
a) Side, b) front, and c) top view (with respect to the cyclopentene five‐membered ring) of the X‐ray crystal structure of anthryl‐fused‐TCBD‐aniline **1**. Carbon atoms are colored in light gray, nitrogen atoms in light blue, and hydrogen atoms in white. Chloroform molecules of crystallization have been omitted for clarity. Structure details are given in the Supporting Information.

Single crystals suitable for X‐ray diffraction analysis were obtained by slow evaporation of a chloroform solution of **1** (Figure 1). As a result of the intramolecular CDA reaction, a bicyclic structure is obtained in which the two “outer” benzene rings of the former anthryl moiety are arranged forming a ∼120° angle between them, and each of them with the five‐member ring (Figure 1b).

**Figure 2:**
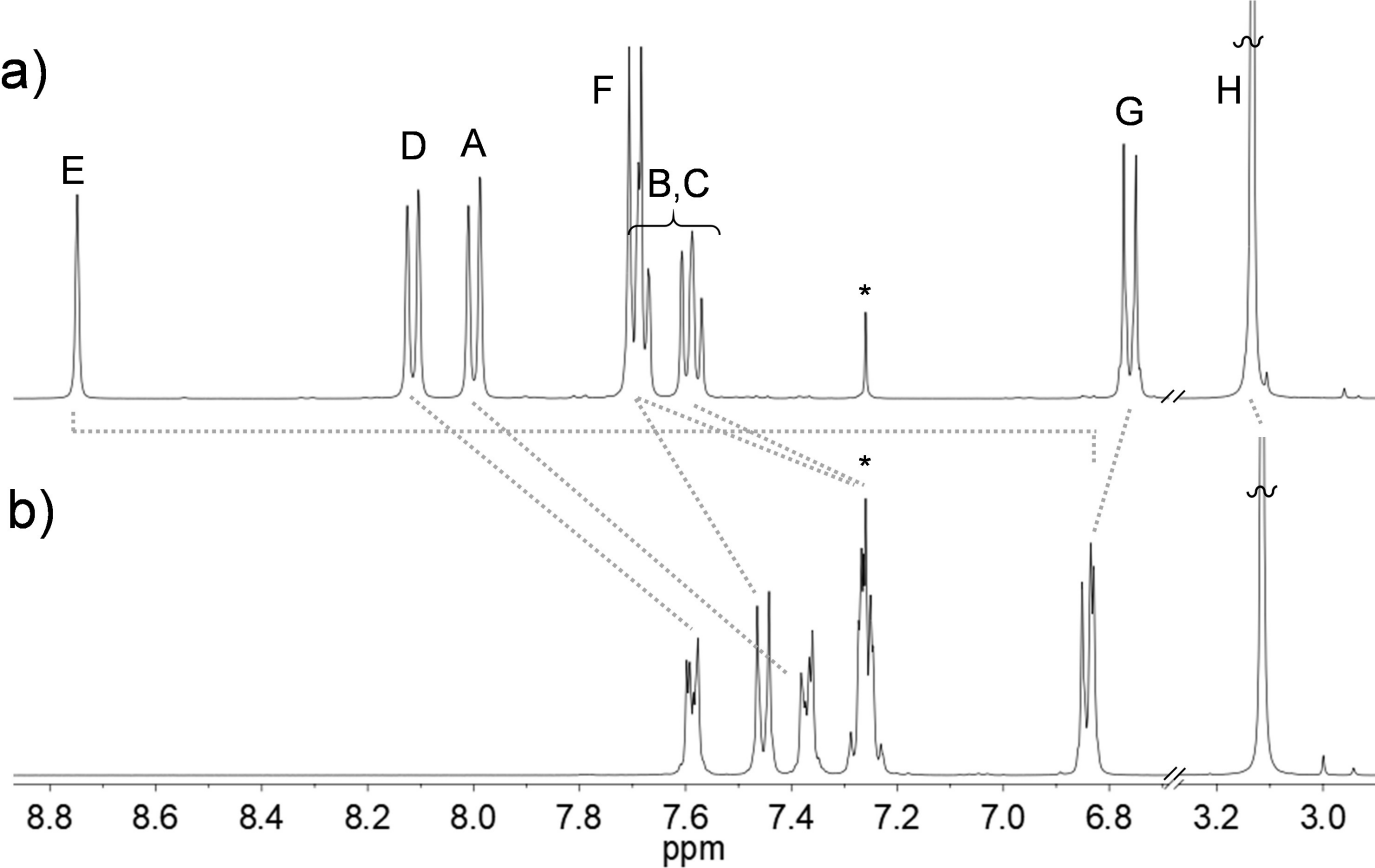
^1^H NMR spectra (CDCl_3_) of a) anthryl‐TCBD‐aniline **2** and b) anthryl‐fused‐TCBD‐aniline **1**. Capital letters refer to protons’ assignment of **1** and **2** in Scheme 1. * denotes residual solvent signals.

The ^1^H NMR spectrum of **1** reveals some striking differences with respect to that of its precursor **2** arising from the geometrical and electronic changes experienced by the anthryl‐fused‐TCBD‐dimethylaniline derivative (Figure 2). In this context, all the aromatic protons in **1** – except the H_G_ methyl protons – experience an upfield shift compared to those of **2**. Among such protons, the most pronounced change, that is, ∼1.9 ppm, is observed for H_E_, due to a change in the hybridization of the carbon atom it is attached to, that is, from sp^2^ to sp^3^, which is accompanied by the “loss” of the aromatic character of the former central anthryl ring.

**Figure 3:**
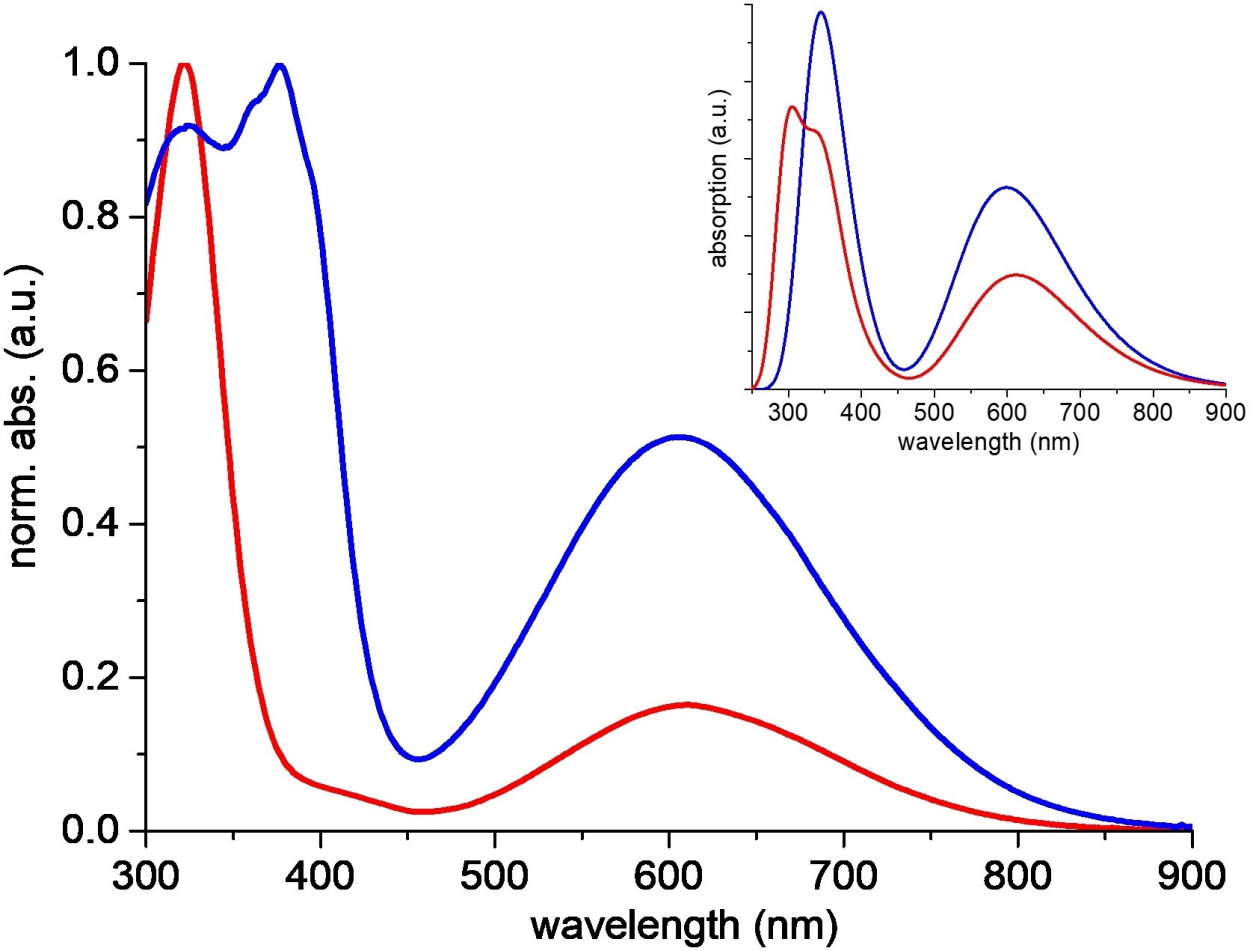
UV‐vis absorption spectra of acetonitrile solutions of **2** (blue line) and **1** (red line). Inset: DFT‐calculated spectra of **2** (blue line) and **1** (red line) in acetonitrile.

Next, the optical properties of anthryl‐fused‐TCBD‐dimethylaniline **1** and its “open” precursor **2** were examined, both experimentally and theoretically. The UV‐vis absorption spectrum of anthryl‐TCBD‐aniline **2** in acetonitrile shows two intense, high‐energy bands at around 325 and 376 nm, and a broader, low‐energy one peaking at 605 nm (*ϵ*=9.7×10^3^ M^−1^ cm^−1^) and tailing into the NIR (blue line in Figure 3). Surprisingly, **2** does not present the typical aniline‐TCBD charge transfer (CT) band at around 450/470 nm typically observed in aniline‐TCBD‐based derivatives (see below).[^4^, ^5^, ^14^, ^18^, ^19^, ^21^, ^22^, ^39^]

Turning to anthryl‐fused‐TCBD‐aniline **1**, a small bathochromic shift ‐ compared to **2** ‐ of the low‐energy transition was observed, that is, from 605 to 611 nm (*ϵ*=5.1×10^3^ M^−1^ cm^−1^), together with a sharp and intense band at around 321 nm (red line in Figure 3).

In order to shed some light onto these peculiar optical properties, quantum mechanical (QM) calculations were carried out at time‐dependent density functional theory (TD‐DFT) level (see section 1 in the Supporting Information). Overall, the computed UV‐vis absorption spectra of **1** and **2** in acetonitrile nicely match the experimental counterparts (inset in Figure 3), and reasonably reproduce the observed hypsochromic shift when going from acetonitrile to a less polar solvent such as THF (Figure S3.1 and Table S3.1). Experimentally, it was also observed, to some extent, a solvatochromism of the UV‐vis absorption of **1** and **2** in four different solvents, though aromatic solvents, that is, toluene and benzonitrile, show a behaviour not entirely due to polarity (Figure S3.2 and Table S3.2).

Theoretical calculations revealed that the broad, low‐energy band of **2** at around 600 nm in acetonitrile has two contributions, the main one from the HOMO→LUMO electronic transition, that is, 593 nm and oscillator strength 0.48, and another from the HOMO‐1→LUMO transition, that is, 626 nm and oscillator strength 0.11. Note that while the LUMO is mostly centered on the TCBD moiety of **2**, HOMO and HOMO‐1 are localized on the aniline and anthryl group, respectively (Figure S3.3a). In contrast, the corresponding low‐energy band of **1** is essentially due to the HOMO→LUMO transition, which, similarly to **2**, corresponds to an electronic rearrangement from aniline, that is, HOMO, to the anthryl‐fused‐TCBD, that is, LUMO, upon excitation (Figure S3.3b). In particular, the extent of the electronic rearrangement upon HOMO→LUMO transition for **1** in acetonitrile was analyzed in terms of the electron density change, showing a character consistent to a CT transition (Figure S3.4).

To better investigate the reason of the “missing” aniline‐TCBD CT band at around 450 nm in **2**, we carried out QM calculations on a model TCBD‐aniline derivative, namely phenyl‐TCBD‐aniline **4** (see structure in the Supporting Information). Derivative **4** presents the typical aniline‐TCBD CT band centered at around 465 nm in THF (Figure S3.6a). The computed UV‐vis absorption spectrum of **4** in THF displays the expected CT band at 471 nm (Figure S3.6b), again characterized by an electronic transition from the aniline‐centered HOMO to a TCBD‐centered LUMO (Figure S3.5). Since we noted a local change in geometry of the aniline group with respect to the TCBD moiety between the phenyl (**4**) and the anthryl (**2**) derivatives (Figure S3.7 and Table S3.3), we investigated whether the observed different optical signature is mainly the result of a structural change or a direct substituent effect, that is, phenyl versus anthryl. To this end, we generated a molecular model of **2** adopting a similar local configuration of the optimized phenyl‐based derivative **4**, that is, same dihedral angles, see Figure S3.7 and caption therein. Interestingly, the recomputed UV‐vis absorption spectrum displays a low‐energy band at around 499 nm, thus recovering the “missing” aniline‐TCBD CT band (Figure S3.6b). By comparing the three absorption spectra in Figure S3.6b, it was concluded that the CT band is shifted in **2** to longer wavelengths, that is, 581 nm, with respect to other TCBD‐aniline derivatives owing to a local geometry change occurring at the TCBD‐aniline fragments, as induced by steric effect of the anthryl group.

**Figure 4:**
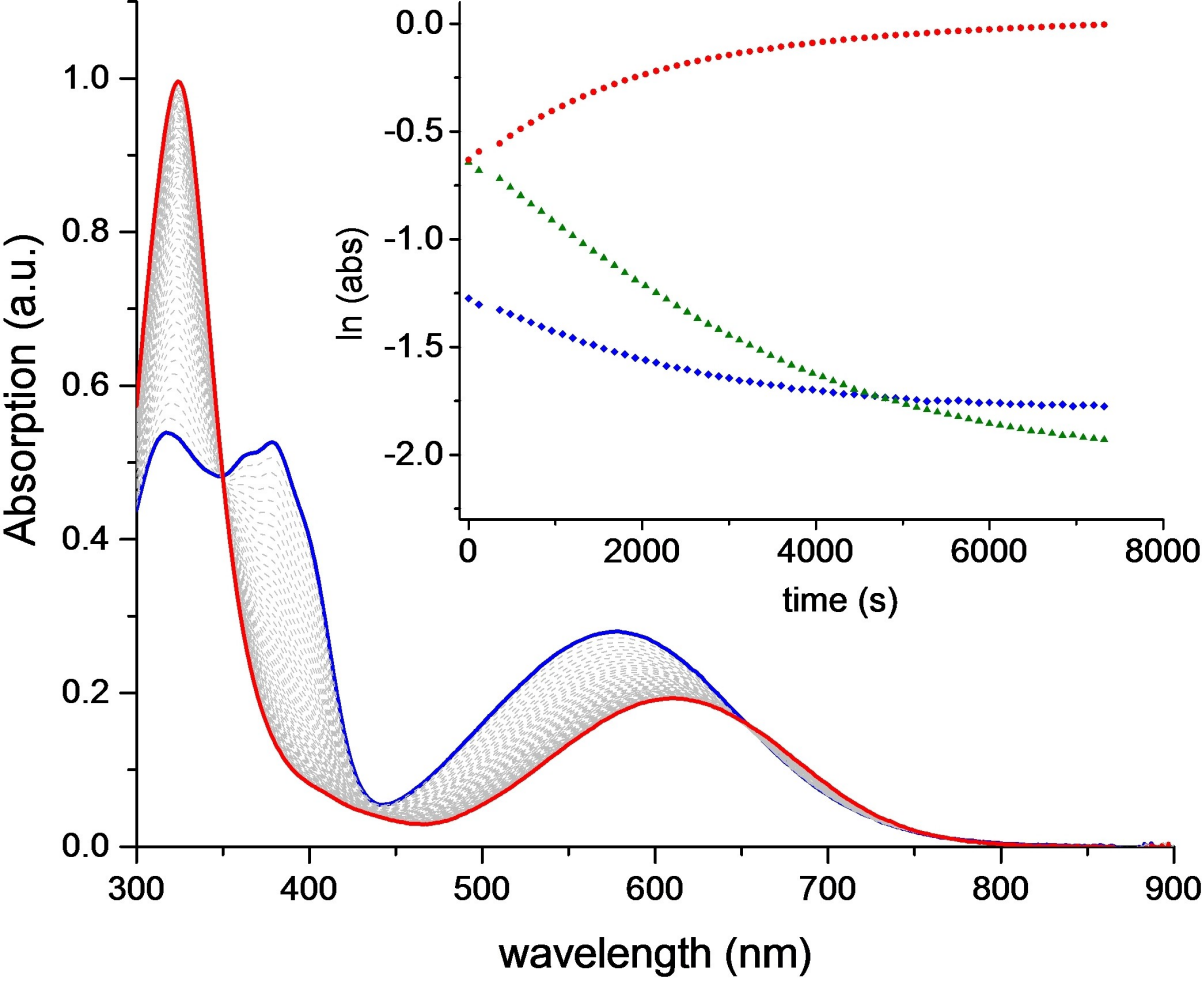
UV‐vis absorption spectra of a toluene solution of **2** heated at 90 °C recorded at *t*=0 (blue line) and *t*=76 min. (red line); intermediate spectra recorded every 2 min (grey lines). The blue and red line spectra correspond to that of anthryl‐TCBD‐aniline and anthryl‐fused‐TCBD‐aniline **2** and **1**, respectively. Inset: Variation of the absorbance (ln abs) monitored at 324 (circles), 378 (squares), and 575 nm (triangles) as a function of time.

Variable‐temperature, that is, 60–90 °C, UV‐vis absorption studies of toluene solutions of **2** allowed to follow its transformation into its “fused” analogue **1** (Figures 4 and S2.15). The presence of two isosbestic points at 653 and 350 nm suggests that the thermally‐promoted conversion of **2** into **1** takes place without the formation of intermediate species. At the highest temperature, that is, 90 °C, the conversion stopped after about one hour (Figure 4), with no appreciable further changes in the UV‐vis spectrum.

Interestingly, an indistinguishable UV‐vis spectrum was obtained when a toluene solution of pristine **1** was heated for prolonged time (Figure S2.14), suggesting that the thermally‐induced conversion from **2** to **1** is quantitative. Interestingly, a dramatic acceleration of the **2**→**1** CDA transformation was observed upon addition of 1 equ of B(C_5_F_5_)_3_ to a toluene solution of **2** (Figure S2.16). The observed boosting in the CDA reaction rate could be explained assuming a coordination of the Lewis acid borane to the lone pair of the cyano groups thus lowering the dienophile LUMO, ultimately favoring the intramolecular cyclization reaction.

To proceed further, the time evolution of the optical absorption ‐ in logarithmic scale, ln(abs) ‐ was recorded at a few selected wavelengths, that is, 324, 378 and 575 nm, showing a non‐linear trend in all cases (insets in Figures 4 and S2.15). The latter result suggests that the **2**→**1** intramolecular CDA reaction is a reversible transformation, though strongly shifted towards the “product” **1**.

Hence, a kinetic model of an equilibrium reaction, that is, with a forward, *k*_**2**→**1**_, and a reverse *k*_**1**→**2**_ rate constant, was adopted to account for the kinetics of the CDA conversion (see section 1 in the Supporting Information). Once fitted, the model provided a good agreement with experimental data (Figure S3.8) and an estimate of the forward rate constant *k*_**2**→**1**_ of 2 to 40×10^−5^ s^−1^ at various temperatures (Table S3.5), with an activation energy of ∼23 kcal/mol. On the other hand, the backward rate constant *k*_**1**→**2**_ and the related thermodynamic equilibrium constant, that is, *K*_eq_=*k*_**2**→**1**_/*k*_**1**→**2**_, could not be evaluated due to the lack of a quantitative measurement of the concentration of **2** at equilibrium, although the ratio of *k*_**2**→**1**_ and *k*_**1**→**2**_ was estimated to be not less than 10^2^.

**Figure 5:**
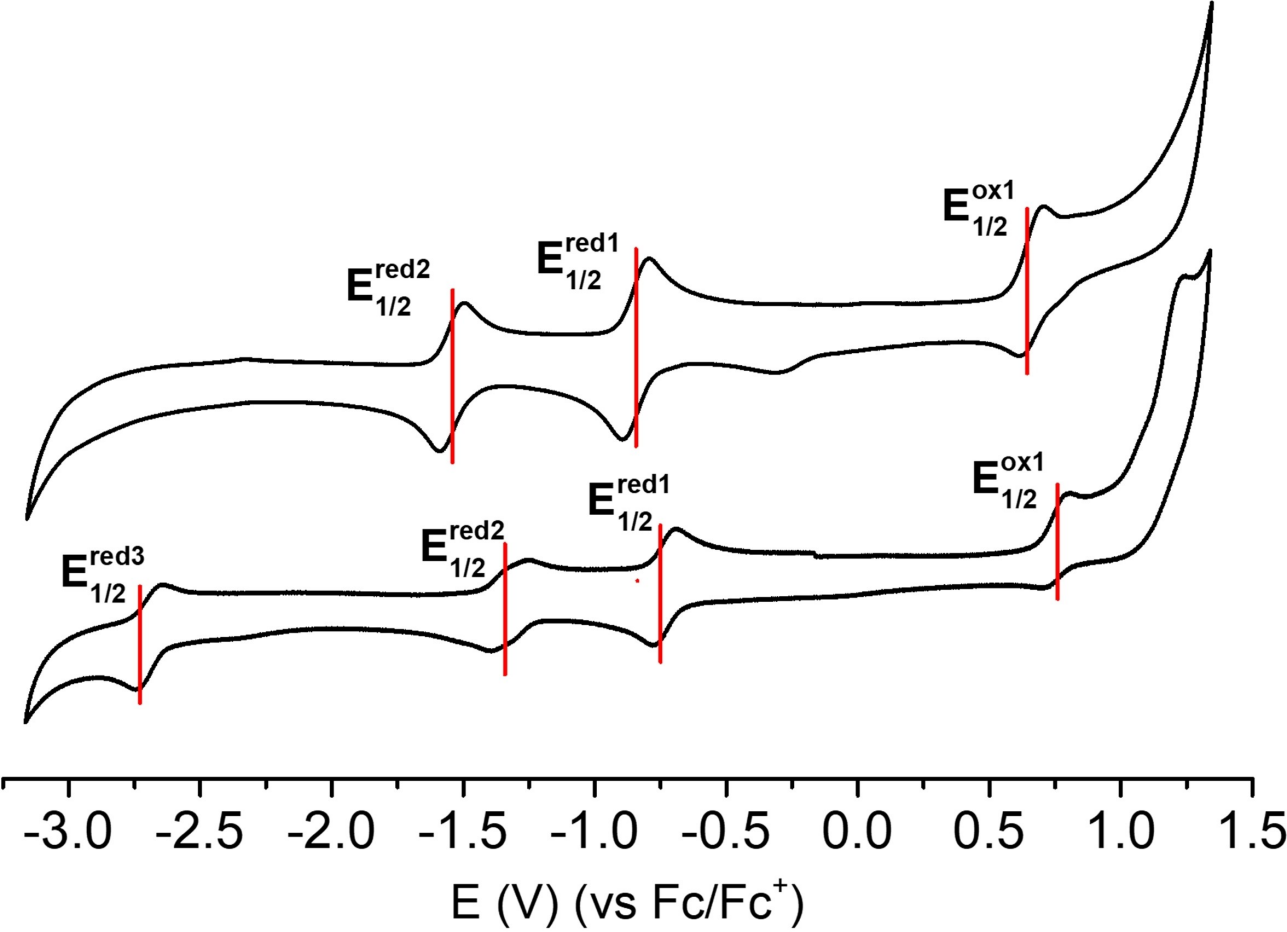
Cyclic voltammograms of **1** (top) and **2** (bottom), measured at a scan rate of 0.1 V s^−1^ in a 0.1 M solution of *n*‐Bu_4_NPF_6_ in THF. Potentials are referred to *E*_1/2_ of the Fc^+^/Fc redox couple.

It is worth noticing that the chemical transformation of one of the four TCBD cyano groups of **2** into an imine in anthryl‐fused‐TCBD‐aniline **1** does not compromise the electron‐accepting capability of the latter derivative with respect to its precursor. In both compounds, several reversible redox peaks are observed, namely two reductions and one oxidation for **1** and three reductions and one oxidation for **2** (Figure 5). In **2**, the three reduction processes occur at −0.75, −1.38, and −2.73 V (versus Fc/Fc^+^), and the oxidation at 0.74 V. The first two reductions are cathodically shifted in **1**, that is, −0.84 and −1.54 V, whereas the oxidation takes place at 0.64 V (Table S4.1). Interestingly, the third reduction process observed in **2** which is occurring at the anthryl moiety,^[40]^ is missing in **1** due to the chemical transformation of the anthryl unit into its TCBD‐fused analog.

UV‐Vis spectroelectrochemistry was performed on derivatives **1** and **2**. For both compounds, the one‐electron reduction and the one‐electron oxidation processes are dominated by a distinct depletion of the aniline‐TCBD CT band at around 600 nm (Figures S4.3 and S4.4). More specifically, oxidation of **1** at 0.98 V (versus Ag wire) generates the aniline radical cation species. The corresponding transformation is characterized by two isosbestic points and new bands with two maxima at 385 (with a shoulder at 450 nm) and 788 nm (Figure S4.3a). Upon reduction of **1** at −0.98 V (vs. Ag wire), an absorption difference spectrum is observed showing a sharp maximum at 471 nm together with a shoulder at 424 nm, which are accompanied by two negative bands at 600 and 725 nm (Figure S4.3b). Oxidation of **2** at 1.0 V (versus Ag wire) results in a minimum at 380 nm and a maximum at 452 nm (Figure S4.4a), while its reduction at −0.83 V (versus Ag wire) leads to an intense band at 450 nm (a shoulder at around 515 nm) along with a weak absorption between 720 and 1000 nm (Figure S4.4b). Superimposed to the broad bleaching of the aniline‐TCBD CT band, a weak feature is discernible at around 628 nm (Figure S4.4b).

Theoretical calculations were carried out to investigate the most important structural and electronic features of **2** and **1** upon reduction. In this case, the HOMOs, as well as the spin density, showed a very similar spatial distribution to the LUMOs of the corresponding neutral forms (Figure S3.9), being essentially located onto the TCBD and fused TCBD moieties. In particular, the central C−C bond, that is, C_1_−C_5_, is shortened by 0.07 Å, while the adjacent bonds, that is, C_1_−C_2_, C_5_−C_6_, are slightly elongated (+0.05 Å) upon reduction (Figure S3.10 and Table S3.5). To obtain a quantitative measure of the electron localization in the reduced species, both compounds were divided into three fragments (see Figure S3.11) and a (Hirschfeld) charge population analysis was performed. As a result, the negative charge (e^−^) was essentially distributed onto the central fragment in both **1** and **2**, thus confirming the strong electron‐accepting capability of the TCBD and fused TCBD moieties in both derivatives.

**Figure 6:**
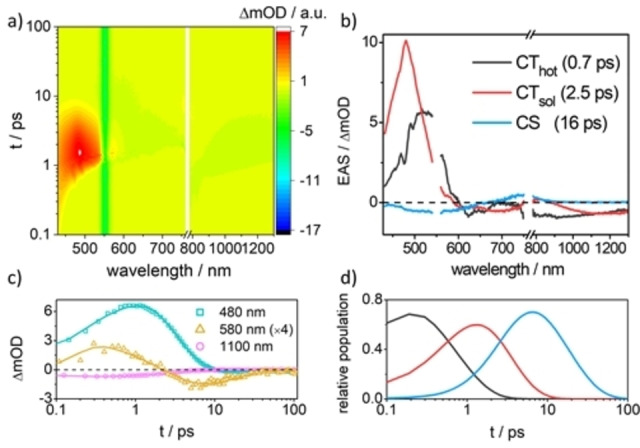
a) Femtosecond transient absorption spectra (fs TAS) of **1** (*λ*_exc_=550 nm) with time delays between 0.1 and 100 ps in the visible and near‐infrared region in air‐saturated benzonitrile solution at room temperature. b) Evolution‐associated spectra (EAS) obtained by a global fit of the fs TA data according to a three‐species kinetic model in GloTarAn.^[42]^ c) Selected kinetic traces with the corresponding fit. d) Concentration evolution with time related to each species in b).

To shed light on the excited‐state dynamics in **1**, femtosecond transient absorption spectroscopy (fs‐TAS) in benzonitrile was performed. Upon photo‐excitation of the CT transition at 550 nm, **1** instantaneously reveals ground‐state bleaching between 600 and 700 nm, which is accompanied by an excited‐state absorption at around 520 nm and a broad stimulated emission from 800 up to 1300 nm (Figure 6a). The excited‐state absorption narrows and blue shifts to 480 nm within 1 ps (Figure 6a and c). Subsequently, the 480 nm maximum decreases in intensity and the broad stimulated emission diminishes entirely at around a time delay of 5 ps (Figure 6c). In **1**, all the excited states decay rapidly and no TAS signal remains after 100 ps.

Quantitative analyses were made through a global fit of the fs‐TAS data according to a sequential three‐species kinetic model. The three lifetimes and the corresponding evolution‐associated spectra in benzonitrile are shown in Figure 6b (see also Figure S5.6a). The first species implies the initially formed vibrationally excited aniline^*δ*+^‐fused TCBD^*δ*−^ CT state (CT_hot_). With a time constant of 0.7 ps, solvent‐induced relaxation occurs leading to the solvent‐relaxed aniline^*δ*+^‐fused TCBD^*δ*−^ CT state (CT_sol_). This assignment is backed up by the blue‐shifting of the excited‐state absorption maximum from, for example, 520 to 480 nm as well as its spectral narrowing. Independent support for assigning the aniline^*δ*+^‐fused TCBD^*δ*−^ CT state came from theoretical and spectroelectrochemical results. The intense 480 nm feature together with two negative bands at 610 and 700 nm manifest the formation of the fused TCBD radical anion (Figure S4.3b). CT_sol_ is short‐lived and converts to the third component in 2.5 ps (Figure 6b). The third component displays a positive feature between 700 and 900 nm, which correlates with the absorptions of the fused TCBD radical anion and the aniline radical cation (Figure S4.3). Additionally, the third species exhibits a weak 443 nm feature, which is buried within the ground‐state bleaching, reflects the formation of the aniline radical cation. Spectroelectrochemistry indicates a weak absorption of the aniline radical cation at 450 nm (Figure S4.3a).^[41]^ Considering that the stimulated emission in the NIR region of the third component is absent, it is attributed to the fully charge‐separated (CS) state aniline^.+^‐fused TCBD^.−^.

When we used 430 nm to populate the locally excited (LE) state, three species were sufficient for globally fitting the fs‐TAS data (Figure S5.1). The evolution‐associated spectra were ascribed to three sequentially populated excited states, that is, the LE state (1.0 ps), aniline^δ+^‐fused TCBD^δ−^ CT state (1.7 ps) and aniline^.+^‐fused TCBD^.−^ CS state (12 ps). The assignments of the latter two species were made according to the very close spectral features and dynamics observed upon photo‐excitation at 550 nm (Figure 6b). It is interesting to point out that the formation of the CS species of **1** is also consistent with our theoretical investigation (see above) (Figure S3.4).

Turning to a less polar solvent such as toluene, a three‐species kinetic model was also employed to analyze the fs‐TAS data (*λ*_exc_=550 nm) (Figures S5.2 and S5.6b). The assignment of the first two species is the same as in benzonitrile, namely CT_hot_ (0.3 ps) and CT_sol_ (3.6 ps). However, in contrast to benzonitrile, the third species in toluene displays an intense excited‐state absorption at 473 nm as well as a stimulated emission in the NIR region (Figure S5.2b, c). The latter points to a species which is different from the CS state generated in benzonitrile. A closer inspection of the differential spectrum suggests the presence of the fused TCBD radical anion (Figures S5.2b and S4.3b). Hence, the third species is assigned to a vibrationally‐relaxed aniline^*δ*+^‐fused TCBD^*δ*−^ CT state (CT_vib_). This assumption is consistent with its blue‐shifted excited‐state absorption from 482 to 473 nm (Figure S5.2b). The absence of the CS state in toluene is likely due to poor stabilization. An increased energy of the CS state is the immediate consequence when contrasting toluene with benzonitrile. This, in turn, would inhibit or significantly slow down the charge separation, which cannot kinetically compete with the structural relaxation process.

Photo‐excitation of **2** yields transient absorption features, which are similar to those described for **1** (Figure S5.6c, d). All the excited states in **2** undergo rapid deactivation within 100 ps (Figures S5.3–S5.5). We applied the same three‐species kinetic model to globally fit the fs‐TAS data (Figures S5.3b–S5.5b). For **2**, the aniline^.+^−TCBD^.−^ CS state is formed as the third species in toluene (Figures S5.3b and S5.6d) and benzonitrile (Figures S5.4b and S5.6c). In both solvents, our assignment is based on i) a weak absorption at around 430 nm, ii) a broad absorption between 700 and 900 nm, and iii) the lack of stimulated emission in the NIR region. Please note that the aforementioned assignments are in sound agreement with the features concluded for the CS state in **1** in benzonitrile. Formation of the CS state in toluene is feasible in **2** due to the slightly higher energy of the CT_sol_ state with 2.08 versus 1.97 eV for **1**.^[43]^ According to our electrochemical assays (Table S4.1), the energies of the CS states in **2** and **1** are 1.49 and 1.48 eV, respectively.^[42]^ Despite the increase in energy of the CS states in toluene, charge separation from CT_sol_ is thermodynamically favored in **2** in contrast to **1**. The CS states decay with 6.2 ps in toluene (Figure S5.3b) and 7.3 ps in benzonitrile (Figure S5.4b) as they return to the ground state. Changing the photo‐excitation to 430 nm to generate a LE state in benzonitrile, charge separation and charge recombination are seen to take place in 1.6 and 7.5 ps, respectively (Figure S5.5b). Thus, in benzonitrile, photo‐excitation of **2** and **1** leads, independently of the excitation wavelength, to the formation of the aniline^.+^–(fused) TCBD^.−^ CS state, which displays with 16 ps a longer lifetime in the latter than in the former (7.3 ps).

## Conclusion

TCBD is an easy‐to‐conjugate, strong electron‐acceptor moiety widely used for the preparation of electroactive conjugates. With the aim of broadening the chemical space of this unit, we presented here the first example of its use in a CDA reaction, an interesting yet poorly explored chemical transformation.

To this end, a derivative was prepared bearing a TCBD unit at the 9‐position of an anthryl group. The judicious relative arrangement of the TCBD ‐in which one of the four nitriles acts as a dienophile‐ and the anthryl ‐the diene‐ moieties allowed for a high‐yielding, intramolecular CDA reaction. The bicyclic structure of the resulting thermally‐stable anthryl‐fused‐TCBD derivative was determined by single crystal X‐ray analysis. A thorough theoretical, electrochemical, and photophysical investigation on this novel derivative demonstrated its remarkable electron‐accepting capability, and interesting ground‐ and excited‐state electronic features. Moreover, a detailed temperature‐dependent kinetic analysis of the CDA reaction converting the anthryl‐TCBD‐based precursor into its anthryl‐fused‐TCBD analogous was carried out suggesting a reversible transformation strongly shifted towards the anthryl‐fused‐TCBD product.

In summary, we reckon that the present study unravels the promising role of TCBD as a “platform” for efficient CDA reactions leading to cycloadducts with remarkable physicochemical properties. Further applications aiming at incorporating the here‐reported anthryl‐fused‐TCBD moiety into novel D−A conjugates are currently being conducted in our laboratories.

## Experimental Section

Experimental details are given in the Supporting Information. Deposition Number(s) 2095823 contain(s) the supplementary crystallographic data for this paper. These data are provided free of charge by the joint Cambridge Crystallographic Data Centre and Fachinformationszentrum Karlsruhe Access Structures service.

## Conflict of interest

The authors declare no competing financial interest.

## Supporting information

As a service to our authors and readers, this journal provides supporting information supplied by the authors. Such materials are peer reviewed and may be re‐organized for online delivery, but are not copy‐edited or typeset. Technical support issues arising from supporting information (other than missing files) should be addressed to the authors.

Supporting Information
