# Supplementary material for: Expanding the Chemical Space of Tetracyanobuta‐1,3‐diene (TCBD) through a Cyano‐Diels‐Alder Reaction: Synthesis, Structure, and Physicochemical Properties of an Anthryl‐fused‐TCBD Derivative
Source: Chemistry. 2021 Oct 12;27(64):16049–55. doi: 10.1002/chem.202103079 (PMC9292653; doi:10.1002/chem.202103079)
Supplement: Supplementary file 1 — Supporting Information [file CHEM-27-16049-s001.pdf]

Chemistry–A European Journal

Supporting Information

Expanding the Chemical Space of Tetracyanobuta-1,3-diene (TCBD) through a Cyano-Diels-Alder Reaction: Synthesis, Structure, and Physicochemical Properties of an Anthryl-fused-TCBD Derivative

Luis M. Mateo, Luca Sagresti, Yusen Luo, Dirk M. Guldi,* Tomas Torres,* Giuseppe Brancato,* and Giovanni Bottari*

Abbreviations:

ACN = acetonitrile; BZN = benzonitrile: COSY = homonuclear correlation spectroscopy; DCM = dichloromethane; DFT = density functional theory; DPV = differential pulse voltammetry; EAS = evolution associated spectra; ESI = electrospray ionization; EtOAc = ethyl acetate; Fc = ferrocene; fs-TA = femtosecond transient absorption; FT-IR ATR = Fourier transform infrared spectroscopy – attenuated total reflectance; HR = high resolution; HSQC = heteronuclear single quantum correlation; MALDI-TOF = Matrix-Assisted Laser Desorption/ Ionization-Time-Of-Flight; MS = mass spectrometry; QM = quantum mechanical; RF = retention factor; TCBD = tetracyanobuta-1,3-diene; THF = tetrahydrofuran; TLC = thin layer chromatography.

Table of contents

1. Materials and Methods 2

Quantum mechanical calculations 4

Kinetic model for the **2→1** transformation 4

2. Synthesis and characterization of anthryl-fused−TCBD−aniline **1** and its precursors **2** and **3** 5

2.1.1. Synthesis and characterization of 9-(4-*N,N*-dimethylaminophenyl)-ethynylanthracene **3**.... 5

2.1.2. Synthesis and characterization of anthryl−TCBD−aniline **2** 6

2.1.3. Synthesis and characterization of anthryl-fused−TCBD−aniline **1** 10

3. Quantum mechanical calculations and UV-vis absorption studies of derivatives **1** and **2** 19

4. Electrochemical and spectroelectrochemical characterization of derivatives **1** and **2** 28

5. Excited state characterization of derivatives **1** and **2** 30

6. References 34

1. Materials and Methods

Chemicals and solvents were purchased from commercial suppliers (Aldrich, Fluka, Strem, Acros and Fischer) and used without further purification. All dry solvents were freshly distilled under argon over an appropriate drying agent before use. Column chromatography was carried out on silica gel Merck-60 (230-400 mesh, 60 Å). Analytical TLC was performed on aluminium sheets precoated with silica gel 60 F-254 from Merck. Preparative TLC plates (20 x 20 cm plates) were purchased from Merck (silica gel-60, 0.5 mm). ^{1}H- and ^{13}C-NMR spectra were recorded with a Bruker Advance 300 MHz instrument, a Bruker DPX 400 MHz instrument or a Bruker Bruker DRX 500 MHz instrument. Chemical shifts values (δ) are referred to tetramethylsilane. Melting points were determined in an Electrothermal IA9000 series instrument oven and are uncorrected.

IR spectra were recorded on a Bruker ALPHA Platinum-ATR system.

ESI MS spectra were obtained in a Bruker MAXIS II spectrometer.

X-Ray crystal diffraction analysis on anthryl-fused−TCBD−aniline **1** was performed on a Bruker Kappa apparatus at 200 K using two-dimensional detector Apex II with Mo kα radiation (λ = 0.71073 A) (CCDC 2095823). The structures were solved by a direct method with the SHELXT program and refined by the SHELXL-2014/7 program.

UV-vis experiments were carried out by using quartz cells with a 1 cm optical path length in a Varian Cary 50 UV spectrophotometer. The extinction coefficients of **1** and **2** in different

solvents were measured at room temperature by using a PerkinElmer Lambda 2 double beam spectrometer. The data were recorded with a slit width of 2 nm and a scan rate of 480 nm/min. Spectroelectrochemistry was performed with a Cary 5000 double-beam spectrometer (Varian, USA) at room temperature. The measurements were conducted in a homebuilt three-electrode thin-layer cell with a path length of 1 mm. The three-electrode setup consists of a platinum mesh working electrode, a platinum wire counter electrode and a silver wire reference electrode. Potentials were applied using a PC-controlled potentiostat (μAutolab III/FRA 2, METROHM). The measurements were carried out in THF (purged with Ar for 5 min) with 0.2 M NBu_4PF_6 as the supporting electrolyte. A single cyclic voltammogram of the sample (scan rate: 0.1 V/s) was recorded first to identify the proper oxidation and reduction potential (vs. Ag wire) to apply for the spectroelectrochemical measurements.

fs-TA spectra were acquired with the HELIOS (0 to 5500 ps) from Ultrafast Systems. The laser source is the Clark MXR CPA2101 Ti:sapphire amplifier (775 nm, 1 kHz, 150 fs pulse width). The excitation pulses of 550 and 430 nm were generated with a noncollinear optical parameter (NOPA, Clark MXR) and small spectral width was ensured by using a bandpass filter (FWHM 10 nm). A white light supercontinuum is generated by focusing a fraction of the fundamental 775 nm onto a 2 mm (Vis: ~420-760 nm) or 1 cm (NIR: 790-1400 nm) sapphire disk. All the measurements were conducted in 2 mm quartz cuvettes at ambient condition with continuous stirring. The optical density of solution was kept at around 0.3 at the excitation wavelength. The power of the pump beam was kept at 200 nJ. The stability of samples were ensured by recording the UV-vis absorption spectrum before and after the transient absorption experiment. Global analysis of the transient absorption data was performed with the GloTarAn software.

Phenyl–TCBD–aniline **4** (Figure S1.1) used as a reference compound was prepared following a reported procedure.[1]

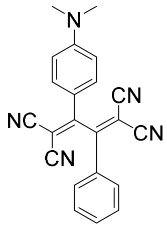

4

Figure S1.1. Molecular structure of phenyl–TCBD–aniline **4**.

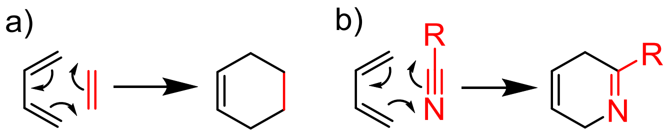

Scheme S1.1. General reaction scheme of a) a Diels-Alder and b) a cyano-Diels-Alder reaction.

Quantum mechanical calculations

Theoretical calculations were performed at DFT level using the Gaussian16 software package.[2] Optimization of the ground state in every system have been done at B3LYP[3]/6-31+G(d,p) level of theory. UV-vis absorption spectra were computed through DFT time-dependent extension (TD-DFT)[4] at CAM-B3LYP[5]/6-31+G(d,p) level of theory. To take care of dispersion energy correction Grimme's D3 dispersion correction[6] has been implemented in all the calculations. In addition, the implicit solvent effect was included by the conductor version of the polarizable continuum model (C-PCM).[5] The crystallographic structures of **1** and **2** were considered as starting point for the QM calculations.

Kinetic model for the 2→1 transformation

The fitting procedure of the rate constants for the **2→1** transformation was reached through the solution in time of the first order reaction close to equilibrium:[7]

$$\frac{[1]}{[1]_0} = \frac{k' + ke^{(k+k')t}}{k + k'}$$

where k is the forward rate constant and k' is the backward rate constant. The dataset used in the fitting has been retrieved from the variation of the absorption peaks (i.e., 324, 378 and 575 nm) through time that, through Lambert-Beer equation, can be related to the variation of the concentration of the reactant and the product in time. These datasets have been produced at five different temperatures (i.e., 333, 343, 348, 353 and 363 K). The forward rate constants have been estimated averaging the fitted ones at different wavelengths at each temperature. The activation energy for the reaction **2→1** has been calculated as the slope of the curve produced by the Arrhenius plot ln(k(T)) *vs* 1/T of the previously computed forward rate constants.

2. Synthesis and characterization of anthryl-fused−TCBD−aniline 1 and its precursors 2 and 3

2.1.1. Synthesis and characterization of 9-(4-*N,N*-dimethylaminophenyl)-ethynylanthracene 3

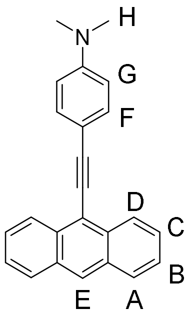

Compound **3** was prepared with a modified procedure from literature:[8]

9-Bromoanthracene (257 mg, 1 mmol), CuI (10 mg, 0.05 mmol, 5 mol%) and $Pd(PPh_3)_4$ (60 mg, 0.05 mmol, 5 mol%) were added subsequently to a flame-dried 30 mL Schlenk tube and subjected to three cycles of vacuum and argon backfilling each. Then, under argon, THF (6 mL, freshly distilled over sodium) and triethylamine (0.84 mL, dried over molecular sieves) were added and the mixture was heated to 70 °C in a silicone oil bath. Then, 4-ethynyl-*N*,*N*-dimethylaniline (175 mg, 1.2 mmol, 1.2 eq) was dissolved in THF (5 mL, freshly distilled over sodium) and added dropwise to the mixture over the course of 1h, after which the mixture was stirred at 70 °C overnight. The reaction mixture was then allowed to reach room temperature and the solvents were removed under reduced pressure. The mixture was subjected to column chromatography (SiO_2 gel, 2:1 *n*-heptanes/$CHCl_3$), where the intensely fluorescent band was collected. The solvents were evaporated under reduced pressure. The resulting crude solid was dissolved in a small amount of $CHCl_3$ (approx. 20 mL), then MeOH (approx. 2 mL) was added, leading to the precipitation of a yellow solid. The so-obtained suspension was filtered, washed with some more MeOH, collected and dried *in vacuo* to give **3** (74 mg, 23%) as a bright yellow solid. ^{1}H- and ^{13}C-NMR data were consistent with those reported in the literature.

2.1.2. Synthesis and characterization of anthryl−TCBD−aniline 2

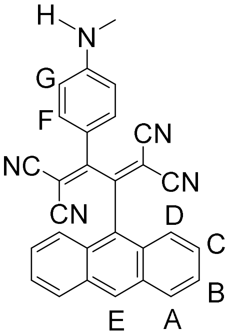

3 (73 mg, 0.227 mmol) was loaded in a 25 mL round-bottomed flask and dissolved in THF (5 mL, freshly distilled over sodium). Then tetracyanoethylene (88 mg, 0.681 mmol) was added and the mixture was stirred at room temperature overnight. The solvent was removed under reduced pressure and the mixture was subjected to column chromatography (SiO_2 gel, eluent = DCM), where the first intense blue band was collected. The solvent was removed under reduced pressure and the crude product was subjected to size exclusion chromatography (BioBeads, $CHCl_3$) and the solvents were then removed under reduced pressure. The resulting crude product was suspended in *n*-hexane, sonicated, filtered and washed with some more *n*-hexane, eventually washed with a few mL of pentane, collected and dried *in vacuo* to yield anthryl−TCBD−aniline **2** (92.6 mg, 91%) as a dark blue solid.

RF = 0.47 (99:1 $CHCl_3$/EtOAc); **^{1}H-NMR** (400 MHz, $CDCl_3$) δ = 8.7 (s, 1H, H_E), 8.1 (d, J = 8.4 Hz, 2H, H_D), 8.0 (d, J = 8.6 Hz, 2H, H_A), 7.8 – 7.7 (m, 4H, H_F+$H_{C/B}$), 7.7 – 7.5 (m, 2H, $H_{C/B}$), 6.8 (d, J = 9.2 Hz, 2H, H_G), 3.1 (s, 6H, H_H); **^{13}C-NMR** (101 MHz, $CDCl_3$) δ = 166.7, 165.4, 154.3, 134.9, 133.2, 131.2, 131.1, 130.2, 129.4, 126.3, 126.2, 123.7, 121.2, 115.0, 112.8, 112.4, 112.3, 111.0, 97.9, 81.8, 40.2; **ESI MS** (positive): m/z 450.1707-452.1737 $[M+H]^{\bullet+}$; **HR MS** (ESI): calcd for $C_{30}H_{20}N_5$ $[M+H]^{\bullet+}$: 450.1713; found: 450.1707; **UV-vis** (THF): λ_{max} (ε) = 585 (9.43×10^3), 379 (1.75×10^4); **FT-IR ATR** ν (cm^{-1}) = 2922, 2855, 2214, 1601, 1485, 1442, 1380, 1200, 1176, 739.

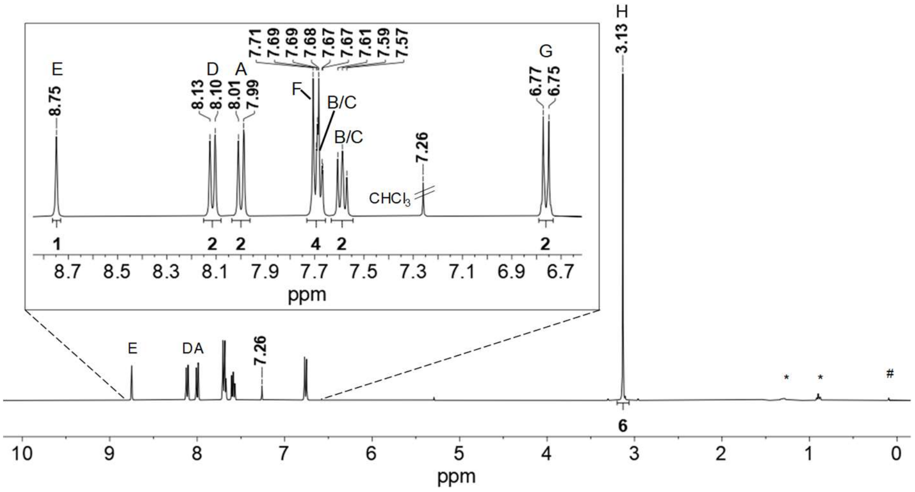

Figure S2.1. ^{1}H-NMR spectrum of anthryl−TCBD−aniline **2** in $CDCl_3$.

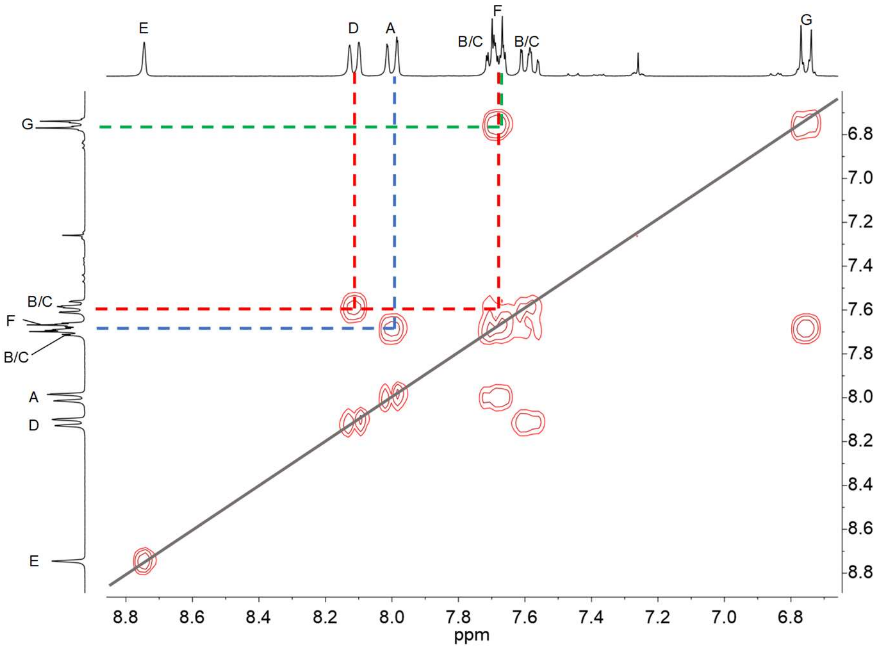

Figure S2.2. ^{1}H-COSY-NMR spectrum of anthryl−TCBD−aniline **2** in $CDCl_3$.

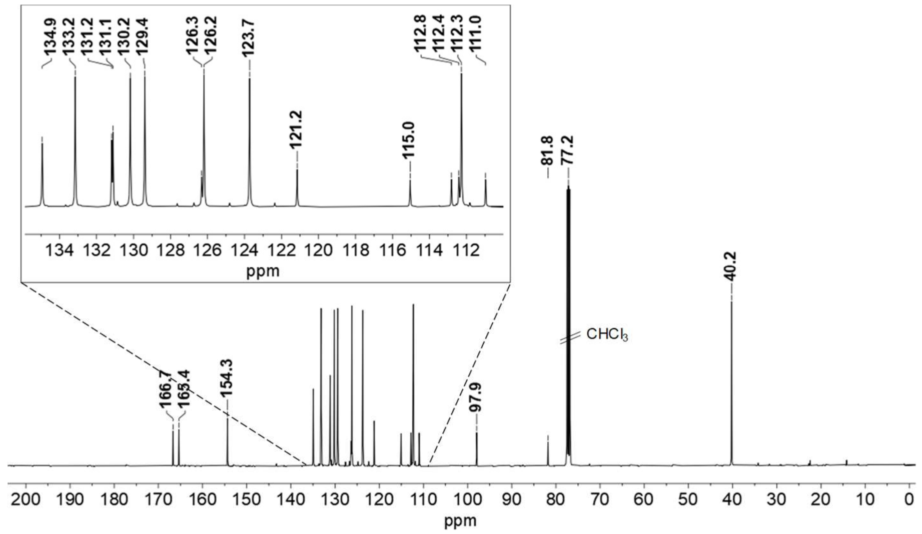

Figure S2.3. ^{13}C-NMR spectrum of anthryl−TCBD−aniline **2** in $CDCl_3$.

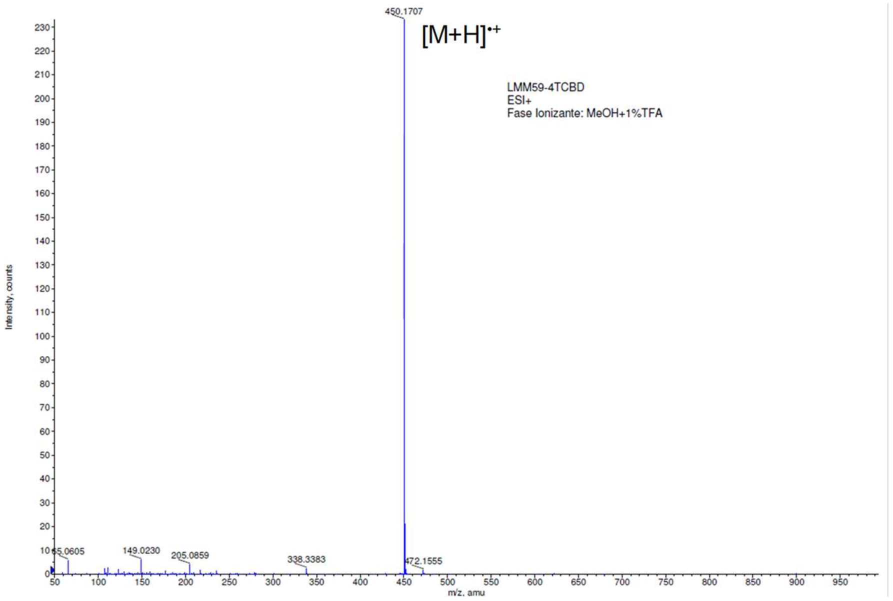

Figure S2.4. ESI mass spectrum of anthryl−TCBD−aniline **2**.

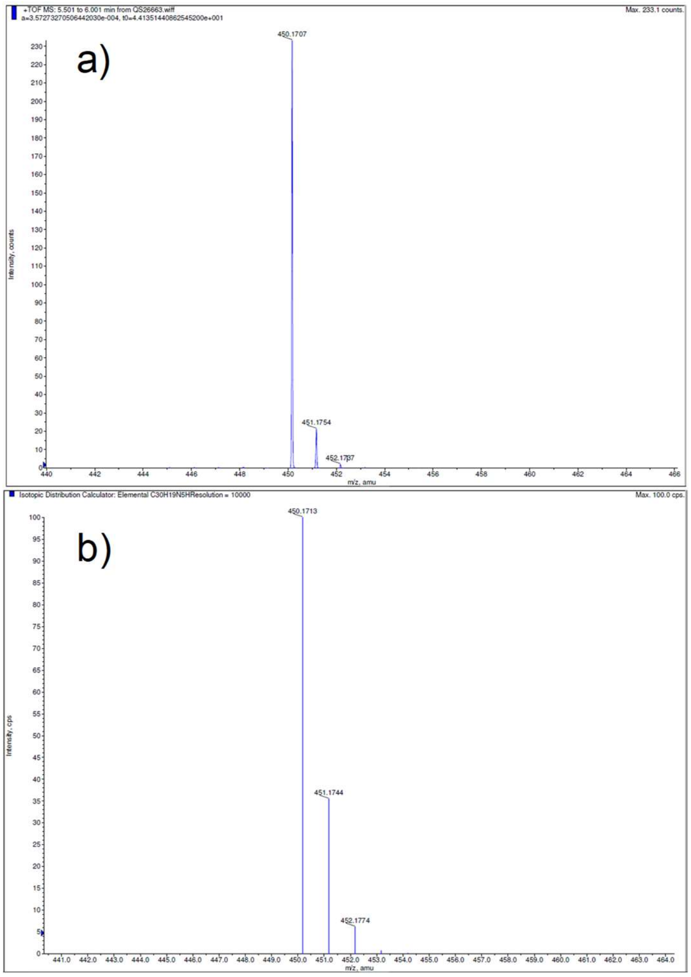

Figure S2.5. ESI HR mass spectrum of anthryl−TCBD−aniline **2**. a) Isotopic distribution of the ESI peaks between 450.1707 and 452.1737 m/z; b) calculated isotopic pattern for anthryl−TCBD−aniline **2**.

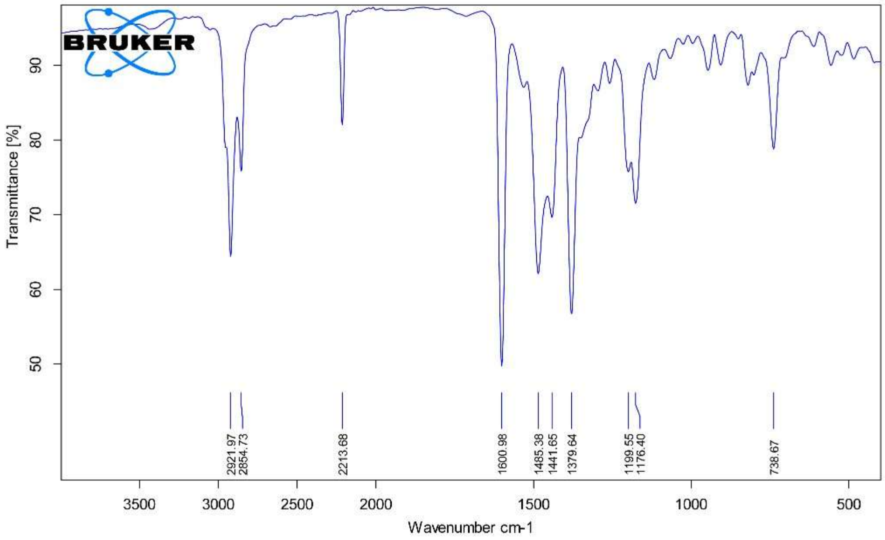

Figure S2.6. FT-IR ATR spectrum of anthryl−TCBD−aniline **2**.

2.1.3. Synthesis and characterization of anthryl-fused−TCBD−aniline 1

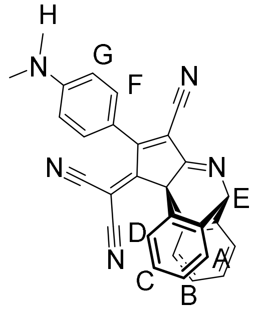

Anthryl−TCBD−aniline **2** (50 mg, 0.11136 mmol) was loaded in a 10 mL round-bottomed flask, dissolved in dry toluene (3 mL) and degassed with argon for several minutes. Then, the reaction was heated to 90 °C and stirred overnight. The solvent was removed under reduced pressure and the mixture was subjected to column chromatography (SiO_2 gel, eluent = DCM to 9/1 DCM:EtOAc), where the second intense blue band was collected. The solvent was removed under reduced pressure and the crude product was subjected to size exclusion chromatography (BioBeads, $CHCl_3$) and the solvents were then removed under reduced pressure. The resulting crude product was suspended in *n*-hexane, sonicated, filtered and washed with some more *n*-hexane, eventually washed with a few mL of pentane, collected and dried *in vacuo* to yield anthryl-fused−TCBD−aniline **1** (38.5 mg, 93%) as a dark blue solid.

RF = 0.85 (95:5 $CHCl_3$/EtOAc); **^{1}H-NMR** (500 MHz, $CDCl_3$) δ = 7.61 – 7.56 (m, 2H, H_D), 7.45 (d, J = 8.9 Hz, 2H, H_F), 7.39 – 7.34 (m, 2H, H_A), 7.29 – 7.22 (m, 4H, H_B+H_C), 6.85 – 6.81 (m, 3H, H_G+H_E), 3.11 (s, 6H, H_H); **^{13}C{^{1}H}-NMR** (101 MHz, $CDCl_3$) δ = 177.3 (C), 164.6 (C), 164.0 (C), 153.1 (C), 143.3 (C), 133.7 (C), 130.9 (CH), 127.7 (CH), 126.8 (CH), 124.8 (CH), 122.4 (CH), 119.4 (C), 114.8 (C), 113.5 (C), 112.3 (C), 111.8 (CH), 110.8 (C), 87.5 (C), 72.4 (CH), 63.3 (C), 40.2 (CH_3); **ESI MS** (positive mode): m/z 450.1702-452.1770 $[M+H]^{\bullet+}$; **HR-MS** (ESI): calcd. for $C_{30}H_{20}N_5$ $[M+H]^{\bullet+}$: 450.1713; found: 450.1702; **UV-vis** (THF): λ_{max} (ε) = 626 (3.77), 327 (4.49); **FT-IR ATR** ν (cm^{-1}) = 2920, 2224, 1601, 1502, 1438, 1369, 1203, 749.

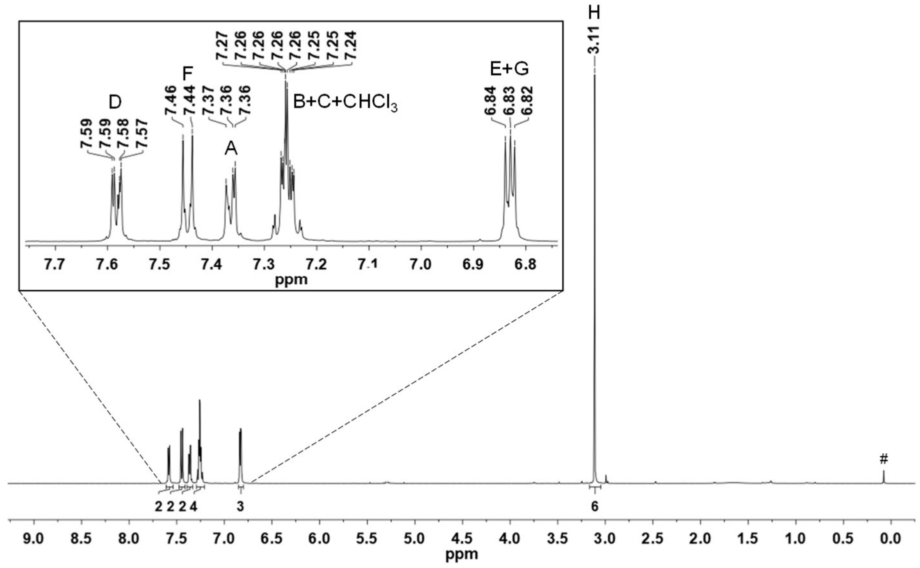

Figure S2.7. ^{1}H-NMR spectrum of anthryl-fused−TCBD−aniline **1** in $CDCl_3$.

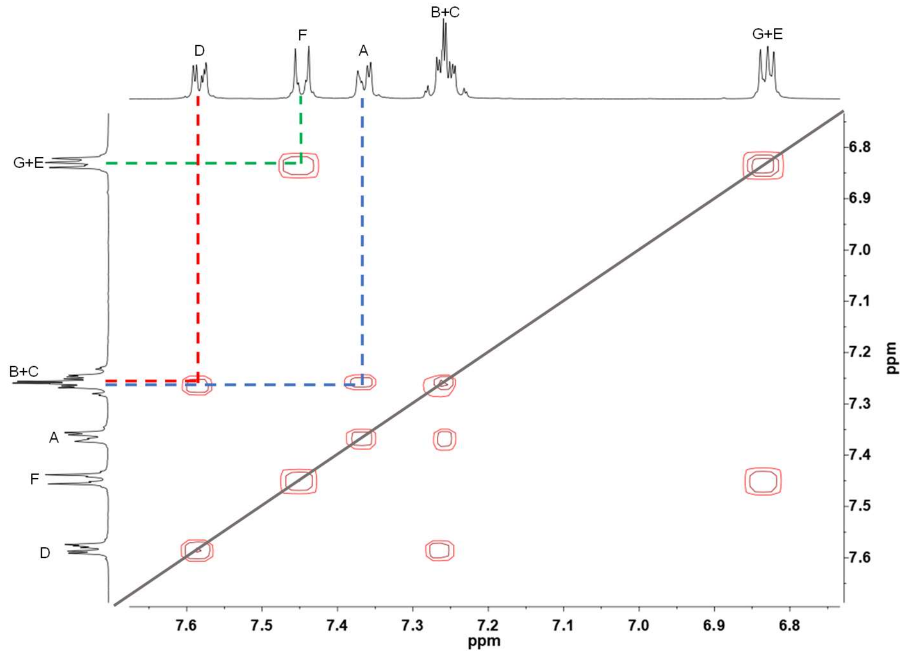

Figure S2.8. ^{1}H-COSY-NMR spectrum of anthryl-fused−TCBD−aniline **1** in $CDCl_3$.

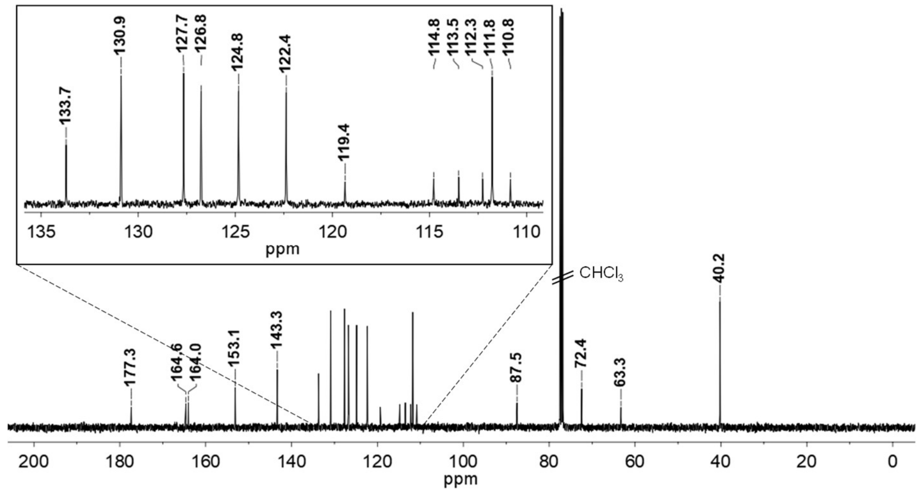

Figure S2.9. ^{13}C-NMR spectrum of anthryl-fused−TCBD−aniline **1** in $CDCl_3$.

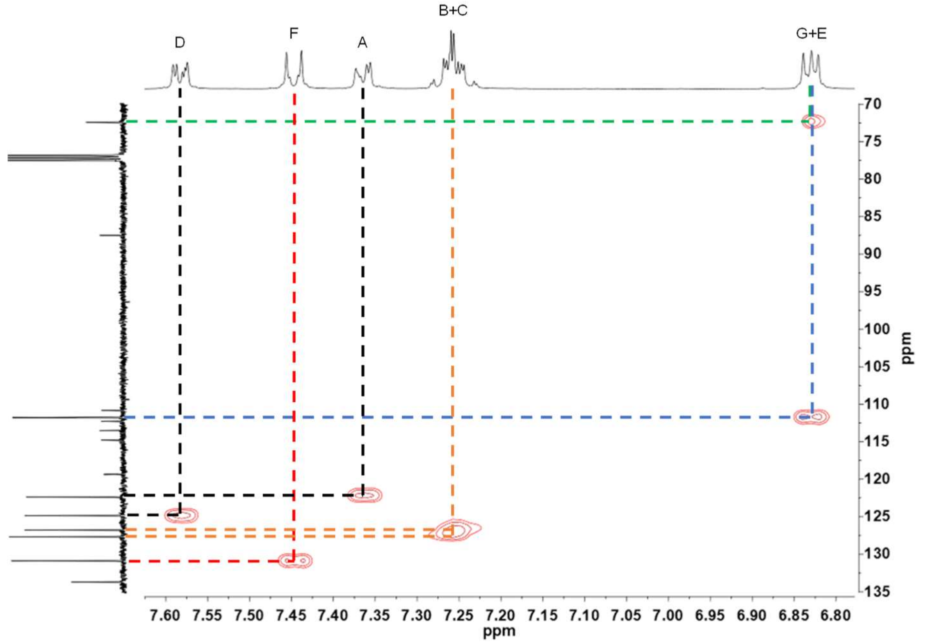

Figure S2.10. $^{13}C\{^1H\}$ HSQC-NMR spectrum of anthryl-fused−TCBD−aniline **1** in $CDCl_3$.

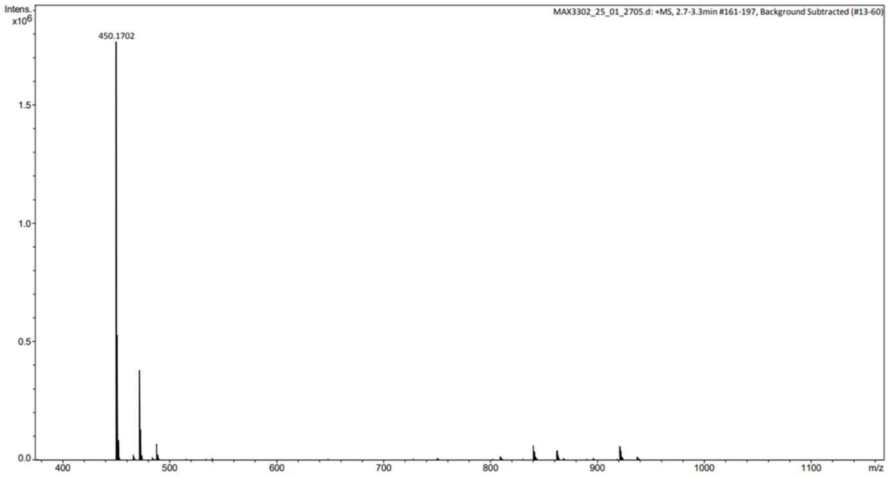

Figure S2.11. ESI mass spectrum of anthryl-fused−TCBD−aniline **1**.

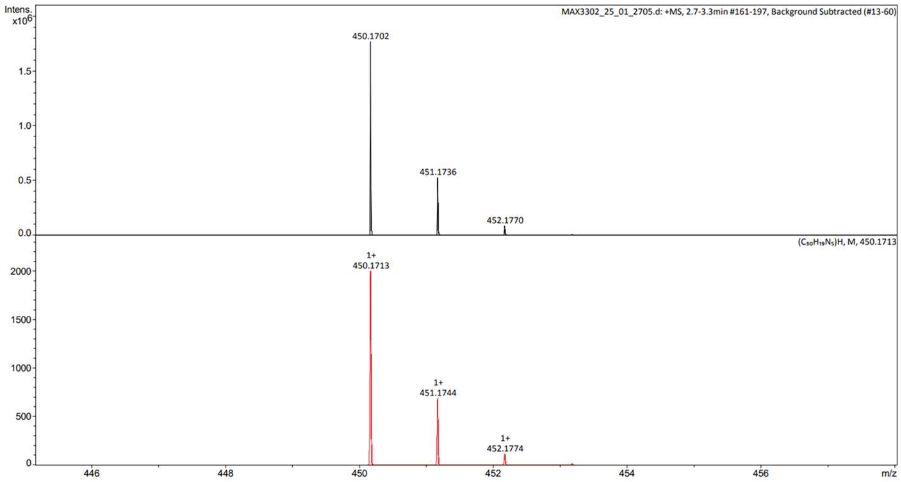

Figure S2.12. ESI HR mass spectrum of anthryl-fused−TCBD−aniline **1**. a) Isotopic distribution of the ESI peaks between 450.1707 and 452.1737 m/z; b) calculated isotopic pattern for anthryl-fused−TCBD−aniline **1**.

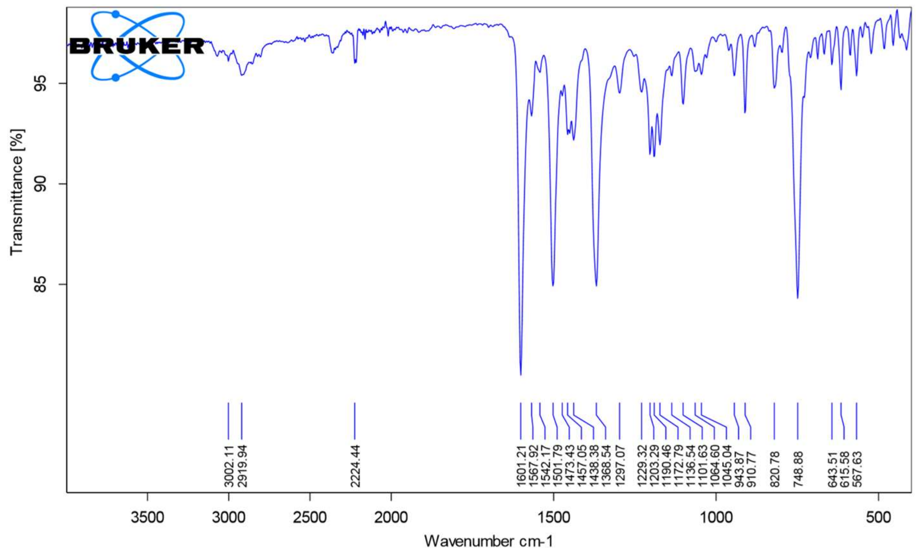

Figure S2.13. FT-IR ATR spectrum of anthryl-fused−TCBD−aniline **1**.

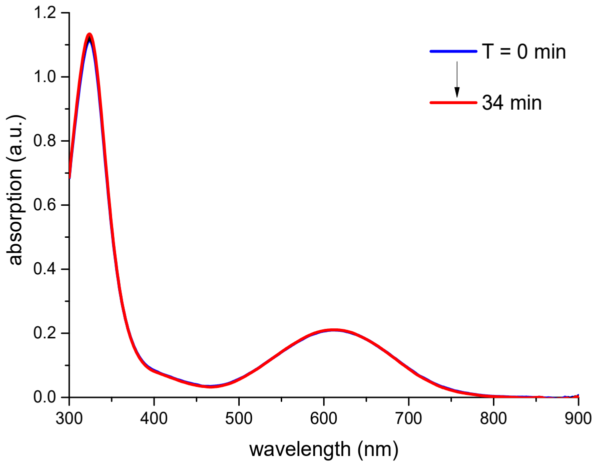

Figure S2.14. UV-vis absorption spectra of a toluene solution of anthryl-fused−TCBD−aniline **1** heated at 90 ºC recorded at *t* = 0 (blue line) and *t* = 36 min. (red line).

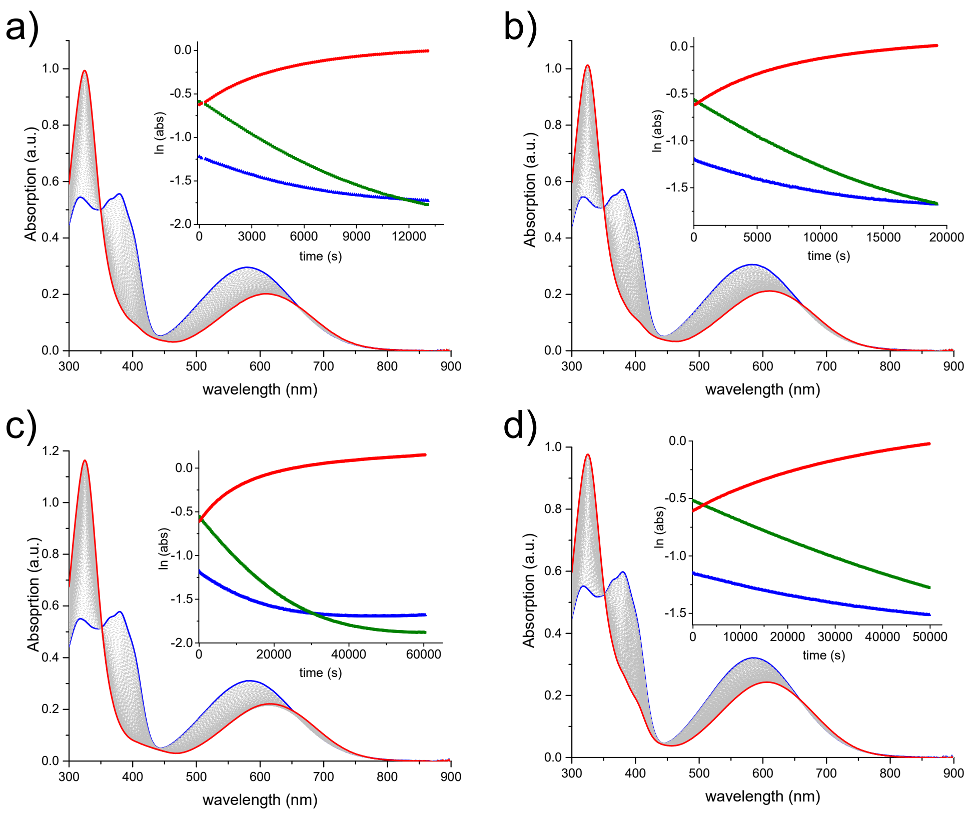

Figure S2.15. UV-vis absorption spectra of toluene solutions of **2** heated at a) 80, b) 75, c) 70, and d) 60 ºC recorded at $t = 0$ (blue line) and a) $t = 218$ min., b) $t = 320$ min., c) $t = 1005$ min., and d) $t = 830$ min. (red line); intermediate spectra (grey lines) were recorded every 2 (a and b) and 5 (c and d) min. The blue and red line spectra correspond to that of anthryl−TCBD−aniline **2** and anthryl-fused−TCBD−aniline **1**. Inset: Variation of the absorbance (ln abs) monitored at 324 (red circles), 378 (blue squares), and 575 nm (green triangles) as a function of time.

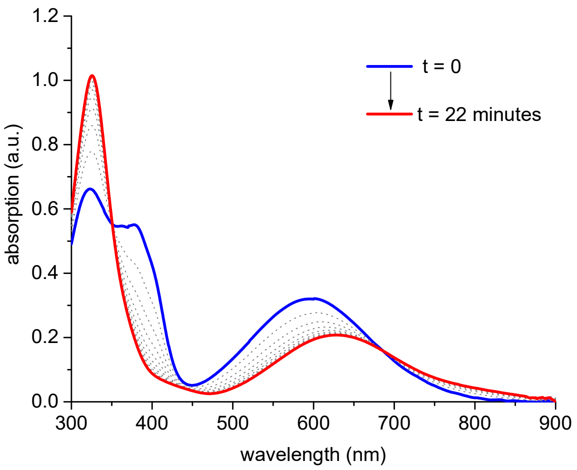

Figure S2.16. UV-vis absorption spectra of a toluene solution of **2** in the presence of 1 equ. of $B(C_5F_5)_3$ heated at 60 ºC recorded at *t* = 0 (blue line) and a) *t* = 22 min.; intermediate spectra (grey dashed lines) were recorded every 2 minutes. The blue and red line spectra correspond to that of anthryl−TCBD−aniline **2** and anthryl-fused−TCBD−aniline **1**. In order to compare the same **2** → **1** CDA transformation at the same temperature (i.e., 60 ºC) but carried out in the absence of $B(C_5F_5)_3$, see Figure S2.15d.

Table S2.1. Selected crystallographic data for anthryl-fused−TCBD−aniline **1**.

Chemical formula	$C_{32}H_{21}Cl_6N_5$	
Formula weight	688.24 g/mol	
Temperature	200(2) K	
Wavelength	0.71073 Å	
Crystal size	0.059 × 0.116 × 0.473 mm	
Crystal habit	dark blue prismatic	
Crystal system	triclinic	
Space group	P-1	
Unit cell dimensions	a = 10.6567(4) Å	α = 71.7044(18)°
	b = 11.3172(5) Å	β = 74.7688(17)°
	c = 14.1867(5) Å	γ = 88.2738(19)°
Volume	1564.86(11) $Å^3$	
Z	2	
Density (calculated)	1.461 g/cm^3	
Absorption coefficient	0.581 mm^{-1}	
F(000)	700	
Theta range for data collection	1.57 to 25.35°	
Index ranges	-12<=h<=12, -13<=k<=13, -17<=l<=17	
Reflections collected	11460	
Independent reflections	5730 [R(int) = 0.0230]	
Coverage of independent reflections	100.0%	
Absorption correction	none	
Max. and min. transmission	0.9670 and 0.7710	
Structure solution technique	direct methods	
Structure solution program	SHELXS-97 (Sheldrick 2008)	

Refinement method	Full-matrix least-squares on F^2	
Refinement program	SHELXL-2014/7 (Sheldrick, 2014)	
Function minimized	$\Sigma\ w(F_o^2 - F_c^2)^2$	
Data / restraints / parameters	5730 / 0 / 391	
Goodness-of-fit on F^2	1.004	
Final R indices	4655 data; I>2σ(I)	R1 = 0.0756, wR2 = 0.2207
	all data	R1 = 0.0903, wR2 = 0.2374
Weighting scheme	$w=1/[\sigma^2(F_o^2)+(0.1437P)^2+3.8004P]$<br>where $P=(F_o^2+2F_c^2)/3$	
Largest diff. peak and hole	1.710 and -0.996 eÅ^{-3}	
R.M.S. deviation from mean	0.109 eÅ^{-3}	

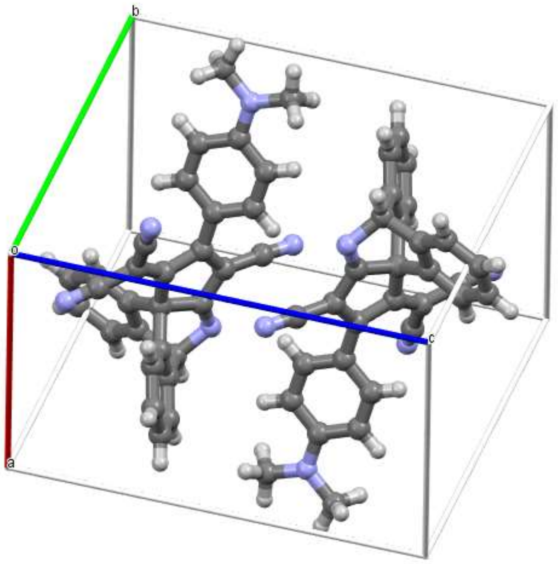

Figure S2.17. View of the unit cell of anthryl-fused−TCBD−aniline **1** crystal structure. Carbon atoms are colored in light gray, nitrogen atoms in light blue, and hydrogen atoms in white. Chloroform molecules of crystallization have been omitted for clarity. The crystallographic axes of the unit cell are colored in red (a axis), green (b axis) and dark blue (c axis).

3. Quantum mechanical calculations and UV-vis absorption studies of derivatives 1 and 2

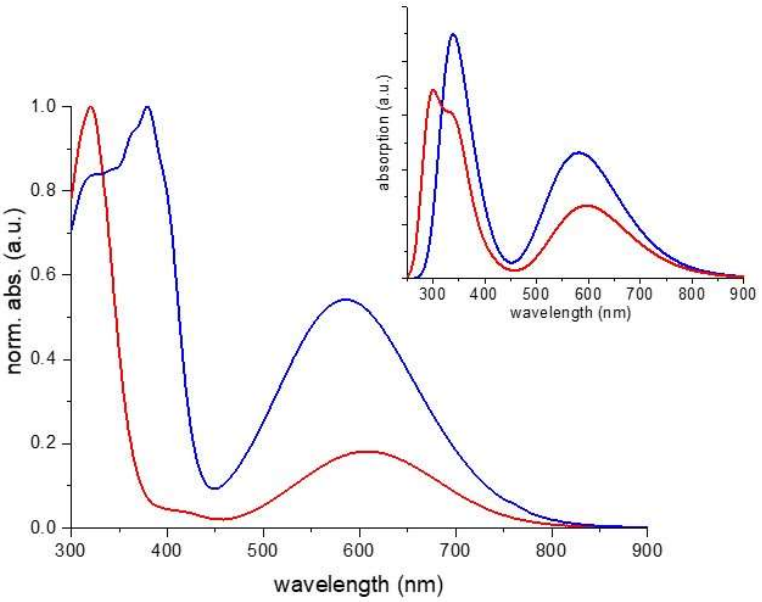

Figure S3.1. UV-vis absorption spectra of THF solutions of **2** (blue line) and **1** (red line). Inset: DFT-calculated spectra of **2** (blue line) and **1** (red line) in THF.

Table S3.1. Experimental and calculated (in parenthesis) peak positions of the low-energy band of **1** and **2** in THF and acetonitrile.

Solvent	λ_{max} (nm) of **1**	λ_{max} (nm) of **2**
THF	609 (596)	586 (581)
acetonitrile	611 (612)	605 (599)

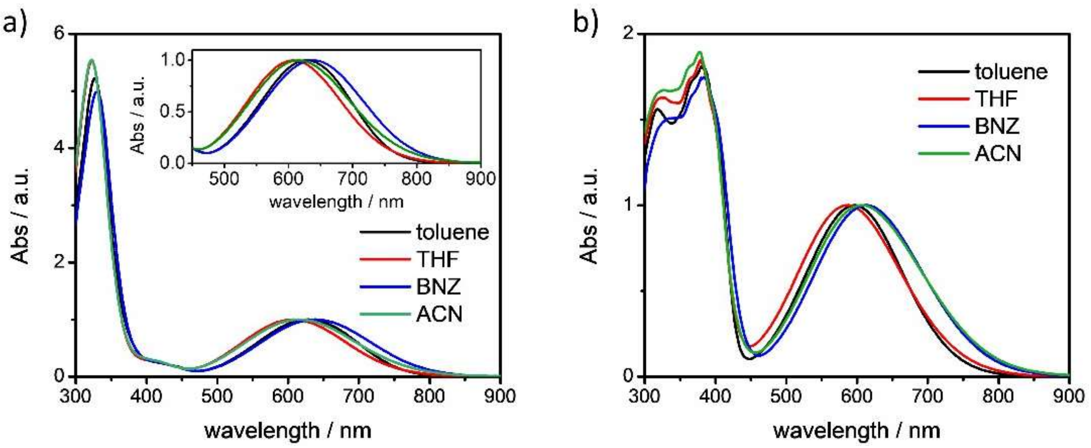

Figure S3.2. Normalized UV-vis absorption spectra of a) **1** and b) **2** recorded in toluene (black line), tetrahydrofuran (red line), benzonitrile (blue line) and acetonitrile (green line). In a) a zoom of the 450-900 nm spectral region is presented.

Table S3.2. UV-vis absorption maxima (λ_{max}) and the corresponding extinction coefficients (Ɛ) for **1** and **2** in different solvents.

compound	λ_{max} (Ɛ) / nm (10^3 M^{-1} cm^{-1})			
	toluene	THF	BNZ	ACN
**1**	326 (29.8)<br>628 (5.8)	323 (29.3);<br>609 (5.3)	329 (27.2);<br>638 (5.5)	322 (28.2)<br>615 (5.1)
**2**	318 (15.8)<br>382 (18.3)<br>596 (10.1)	320 (14.5)<br>379 (16.4)<br>586 (8.9)	331 (15.4)<br>382 (17.6)<br>612 (10.3)	322 (16.2)<br>378 (18.3)<br>605 (9.7)

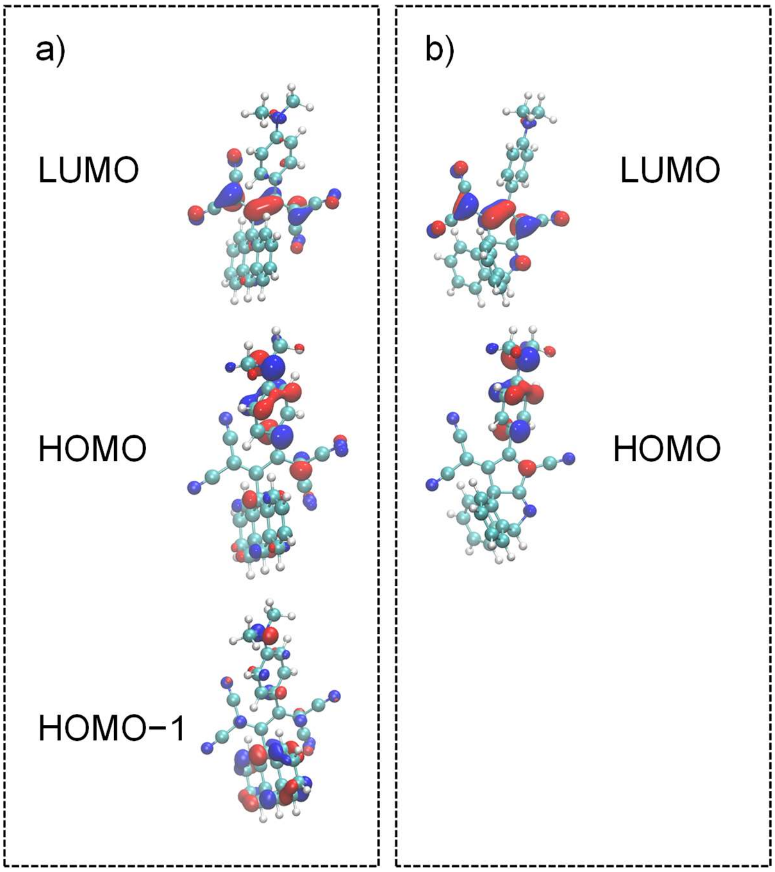

Figure S3.3. Representation of some MOs of a) anthryl−TCBD−aniline **2** and b) anthryl-fused−TCBD−aniline **1** in acetonitrile.

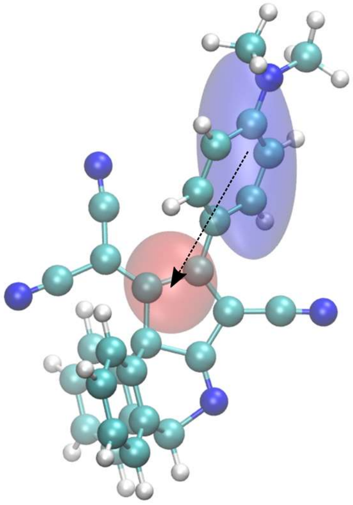

Figure S3.4. Upon HOMO→LUMO electronic excitation of **1** in acetonitrile, the electron density undergoes a decrease at the aniline moiety (i.e., blue centroid region) and an increase at the TCBD unit (i.e., red centroid region). The arrow shows the distance between the two centroids, whose magnitude is 4.05 Å. Computed electron and hole centroids as defined in [[9]].

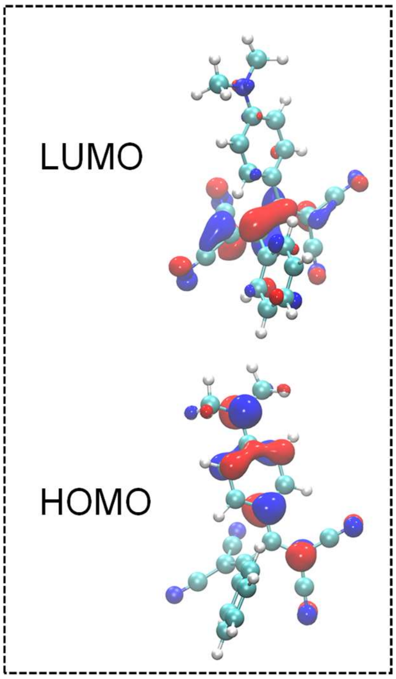

Figure S3.5. Representation of some MOs of phenyl−TCBD−aniline **4**.

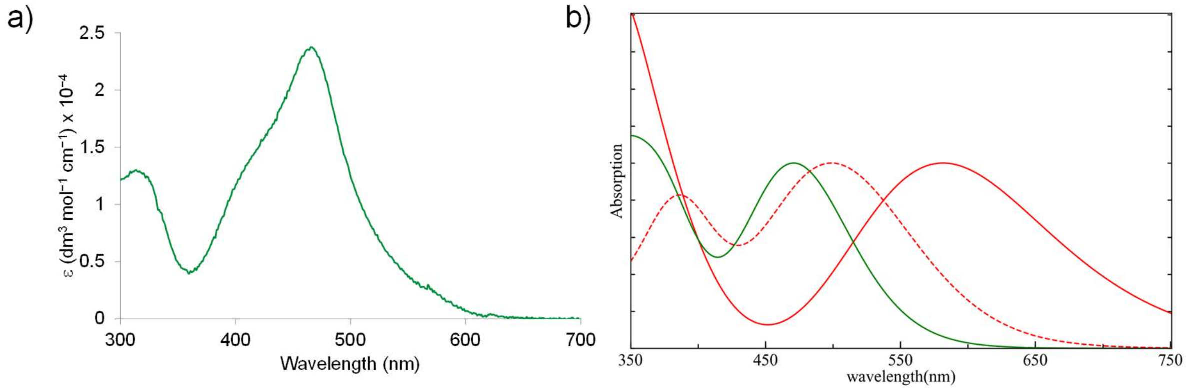

Figure S3.6. a) UV-vis absorption spectrum of a THF solution of phenyl−TCBD−aniline **4**. b) Computed (TD-DFT) UV-vis absorption spectra of anthryl−TCBD−aniline **2** (red solid line) and phenyl−TCBD−aniline **4** (green solid line) in THF. The red dashed line represents the absorption spectrum of anthryl−TCBD−aniline **2** in THF with the dihedral angles kept fixed at values similar to the ones of optimized phenyl−TCBD−aniline **4**. For the sake of comparison, the spectra are scaled with respect to the low-energy peak height.

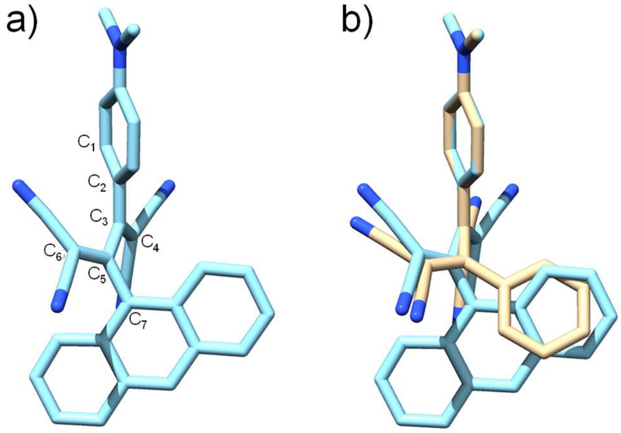

Figure S3.7. a) Optimized structure of anthryl−TCBD−aniline **2** with selected carbons as reference for inspect significant dihedral angles. b) Superposition of anthryl−TCBD−aniline **2** optimized structure in cyan and phenyl−TCBD−aniline **4** in yellow. The match showed an RMSD of 0.007 for the aniline head. The figure emphasizes the different geometry of the TCBD moiety of the two compounds.

Table S3.3. Representative dihedral angles differences between anthryl−TCBD−aniline **2** and phenyl−TCBD−aniline **4**. The notation used for dihedral angles refers to those reported in Figure S3.7.

Dihedral angles	Anthryl−TCBD−aniline **2**	Phenyl−TCBD−aniline **4**
D1 (C_1-C_2-C_3-C_4)	39.1	22.5
D2 (C_1-C_2-C_3-C_5)	−142.1	−158.3
D3 (C_2-C_3-C_5-C_6)	35.5	70.3

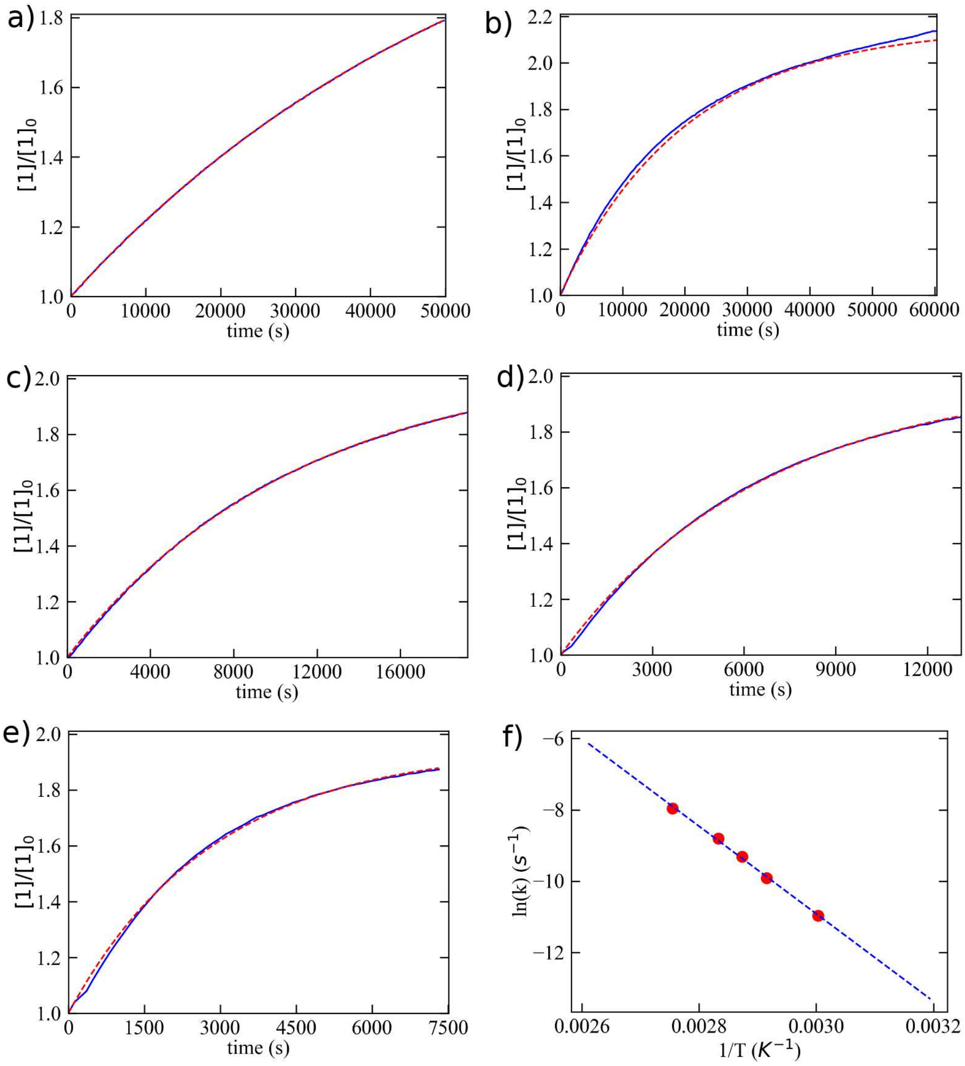

Figure S3.8. Time evolution of the absorption intensity recorded at 324 nm during the **2→1** CDA reaction at increasing temperature: a) 333 K, b) 343 K, c) 348 K, d) 353 K, e) 363 K. The blue lines represent the experimental data and the red dashed lines are the best fit of the kinetic model (see "Kinetic model" section). Similar fitting were obtained for absorption data recorded at 378 nm and 575 nm (data not shown). f) Arrhenius plot of the averaged (from 3 different wavelengths: 324, 378, and 575 nm) fitted forward rate constants *k* as a function of the temperature. Red dots are the computed rate constants (at 333, 343, 348, 353 and 363 K) and the blue dashed line is the linear fit. From the slope of this curve, the activation energy (E_a) for the reaction **2→1** has been estimated.

Table S3.4. Fitted values for the forward rate constant at different temperatures. The mean has been computed using the data from three different wavelengths (324, 378 and 575 nm). Errors are presented as one standard deviation. Linear fit of the logarithm of the computed forward

rate constants *vs* 1/T (Arrhenius plot) gives the value of the activation energy barrier (*E*a) for the reaction **2→1**. The estimated slope for *E*a is 22.9 ± 1.1 kcal/mol.

Temperature (K)	$k_{2\rightarrow1}$ (s^{-1} × 10^{-5})
333	2.3 ± 0.5
343	8 ± 1
348	11 ± 2
353	17 ± 2
363	40 ± 2

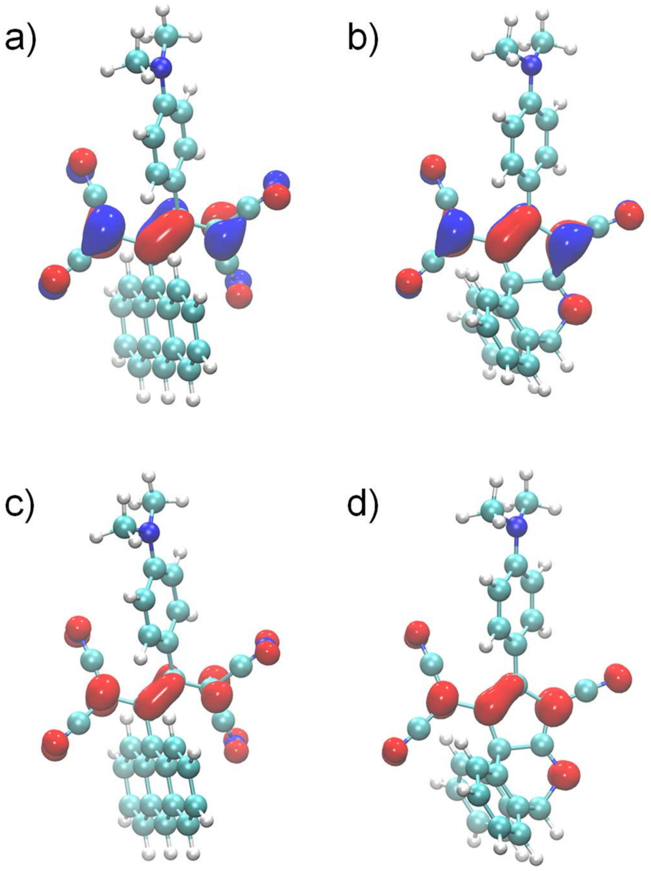

Figure S3.9. Single-occupied molecular orbital (SOMO) and spin density of the anionic forms of a, c) **2** and b, d) **1**, respectively.

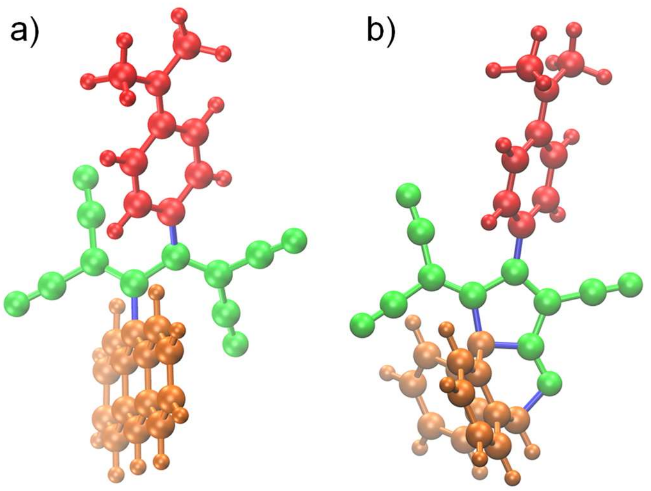

c)

	Red fragment	Green fragment	Orange fragment
Neutral **1**	0.21e	–0.34e	0.13e
Monoreduced **1**	0.04e	–0.99e	0.05e
Neutral **2**	0.37e	–0.51e	0.14e
Monoreduced **2**	0.18e	–1.08e	−0.10e

Figure S3.10. Division of a) anthryl−TCBD−aniline **2** and b) anthryl-fused−TCBD−aniline **1** in their three constituent parts. Red, green and orange colored atoms indicate aniline, multicyano (i.e., TCBD or fused-TCBD) and anthryl moieties respectively. This division into three fragments has been adopted to perform the fragment population analysis of systems **2** and **1** both in neutral and monoanionic form. c) Table summarizing the fragment-based charge population analysis for the neutral and reduced species of **1** and **2**.

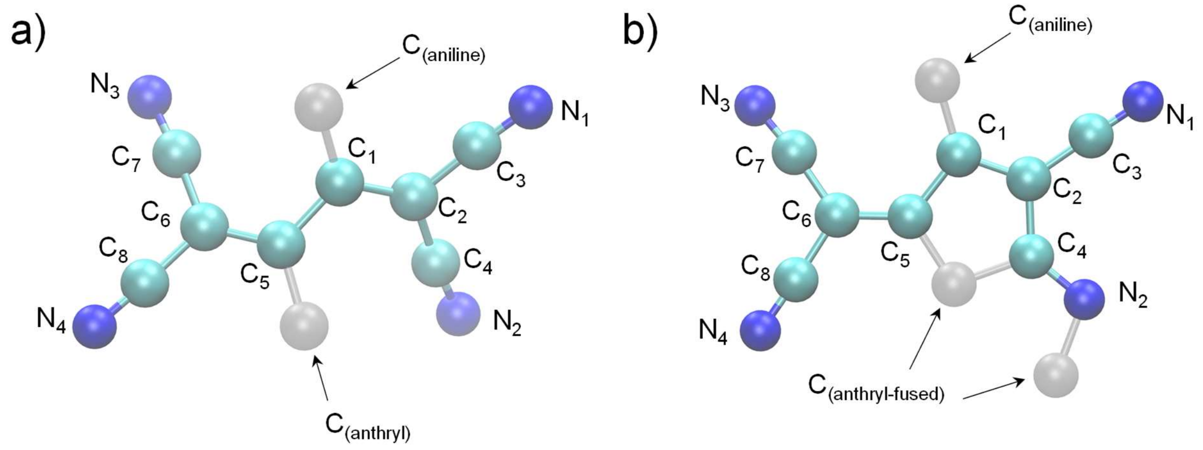

Figure S3.11. Atoms' nomenclature to assess geometrical differences of multicyano moiety (i.e., TCBD or fused-TCBD) in neutral and reduced a) anthryl−TCBD−aniline **2** and b) anthryl-fused−TCBD−aniline **1**.

Table S3.5. Bond lengths for the multicyano moiety (i.e., TCBD or fused-TCBD) for neutral and mono-reduced species of **2** and **1**. The notation used for the bonds refers to those reported in Figure S3.11.

Bond (Ang)	Neutral **2**	Mono-reduced **2**	Neutral **1**	Mono-reduced **1**
C_1-C_2	1.38	1.43	1.37	1.43
C_2-C_3	1.42	1.42	1.42	1.42
C_3-N_1	1.17	1.17	1.17	1.17
C_2-C_4	1.42	1.42	1.42	1.42
C_4-N_2	1.17	1.17	1.17	1.17
C_1-C_5	1.48	1.41	1.47	1.41
C_5-C_6	1.39	1.43	1.39	1.43
C_6-C_7	1.42	1.42	1.42	1.42
C_7-N_3	1.17	1.17	1.17	1.17
C_6-C_8	1.42	1.42	1.42	1.42
C_8-N_4	1.17	1.17	1.17	1.17

4. Electrochemical and spectroelectrochemical characterization of derivatives 1 and 2

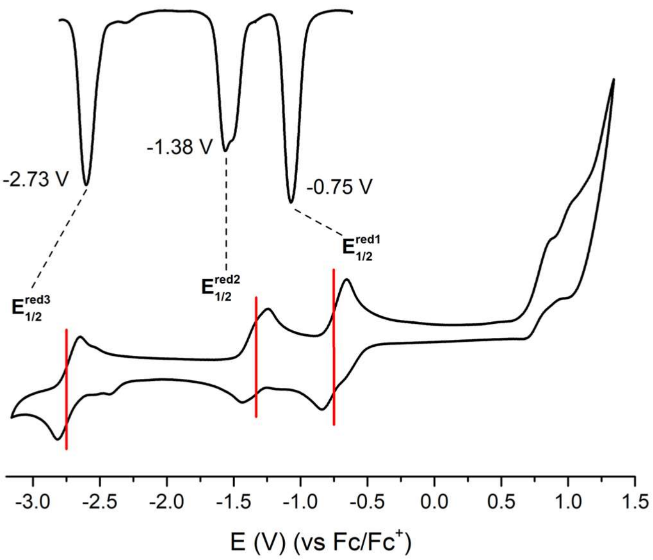

Figure S4.1. Cyclic (bottom) and square wave (top) voltammograms of anthryl−TCBD−aniline **2** at a scan rate of 0.1 V s^{-1} in a 0.1 M solution of *n*-Bu_4NPF_6 in THF. Potentials are referred to $E_{1/2}$ of the Fc^+/Fc redox couple.

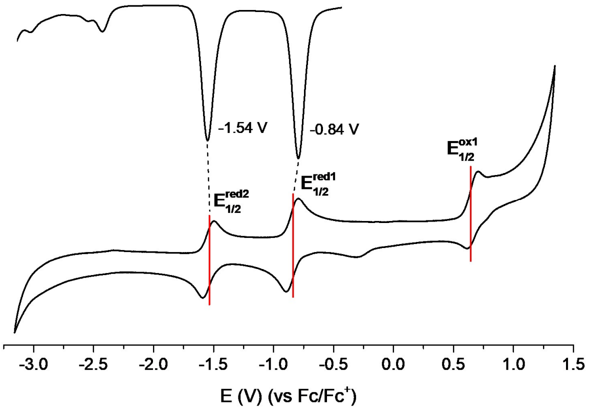

Figure S4.2. Cyclic (bottom) and square wave (top) voltammograms of anthryl-fused−TCBD−aniline **1** at a scan rate of 0.1 V s^{-1} in a 0.1 M solution of *n*-Bu_4NPF_6 in THF. Potentials are referred to $E_{1/2}$ of the Fc^+/Fc redox couple.

Table S4.1. Electrochemical data (*vs.* Fc/Fc^+) of **1** and **2** in THF as determined by DPV.

Compound	$E_{1/2}^{ox,1}$ (V)	$E_{1/2}^{red,1}$ (V)	$E_{1/2}^{red,2}$ (V)	$E_{1/2}^{red,3}$ (V)
**1**	0.64	−0.84	−1.54	
**2**	0.74	−0.75	−1.38	−2.43

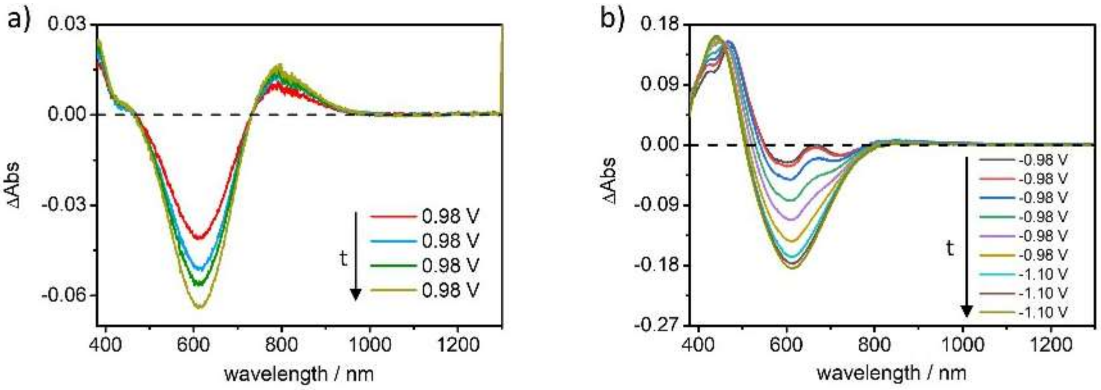

Figure S4.3. Time-dependent absorption difference spectra of the one-electron a) oxidized and b) reduced anthryl-fused−TCBD−aniline **1** recorded in an Ar-saturated THF solution with 0.2 M NBu_4PF_6 as the supporting electrolyte. Potentials are referred to the Ag wire reference electrode. For the reduction process of the anthryl-fused−TCBD moiety, the absorption difference spectra undergo a spectral evolution before reaching a stable shape. As time progresses, the 424 and 471 nm features narrow and merge into a single feature centered at 440 nm, meanwhile the ratio at 600-to-725 nm increases reaching a single minimum at 613 nm. This could be ascribed to a (partial) retro-CDA reaction considering that the spectra recorded at longer time show features similar to that of **2** upon reduction (Fig. S4.4b). Note: both reduction potentials at −0.98 and −1.10 V aim at the first electron reduction process.

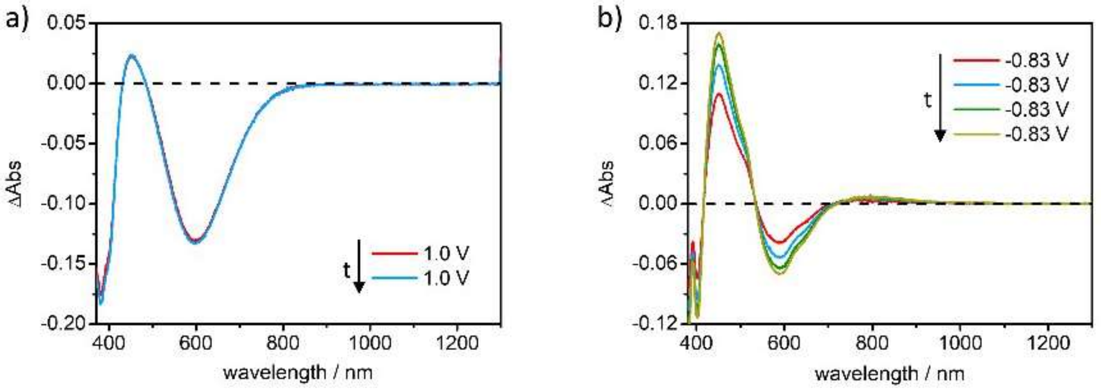

Figure S4.4. Time-dependent absorption difference spectra of the one-electron a) oxidized and b) reduced anthryl−TCBD−aniline **2** recorded in an Ar-saturated THF solution with 0.2 M NBu_4PF_6 as the supporting electrolyte. Potentials are referred to the Ag wire reference electrode.

5. Excited state characterization of derivatives 1 and 2

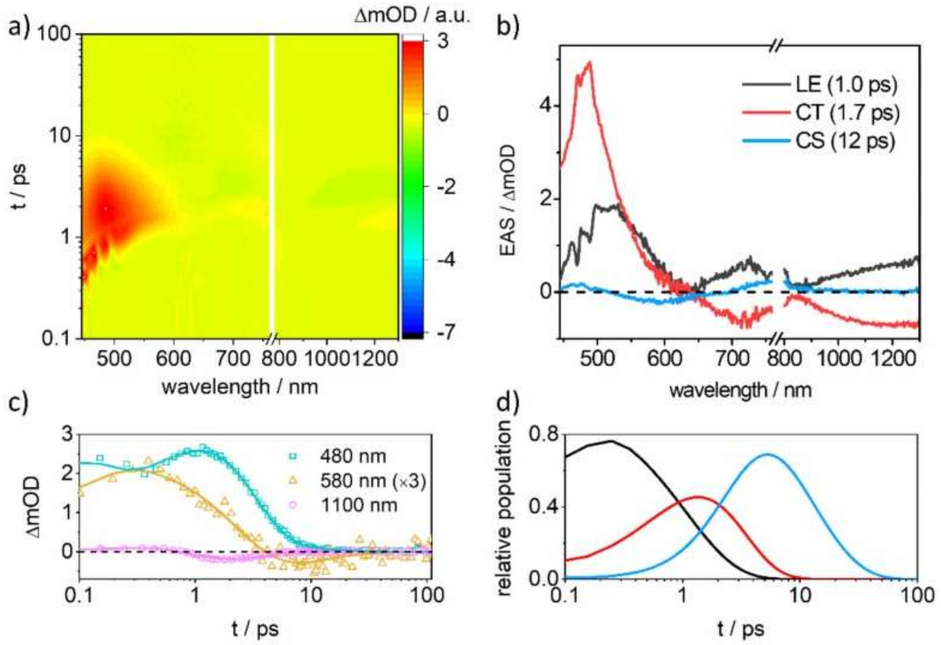

Figure S5.1. a) fs-TAS of **1** (λ_{exc} = 430 nm) with time delays between 0.1 and 100 ps in the visible and near-infrared region in an air-saturated benzonitrile solution at room temperature. b) EAS obtained by a global fit of the fs-TAS data according to a three-species kinetic model in GloTarAn. c) Selected kinetic traces with the corresponding fit. d) Concentration evolution with time related to each species in b).

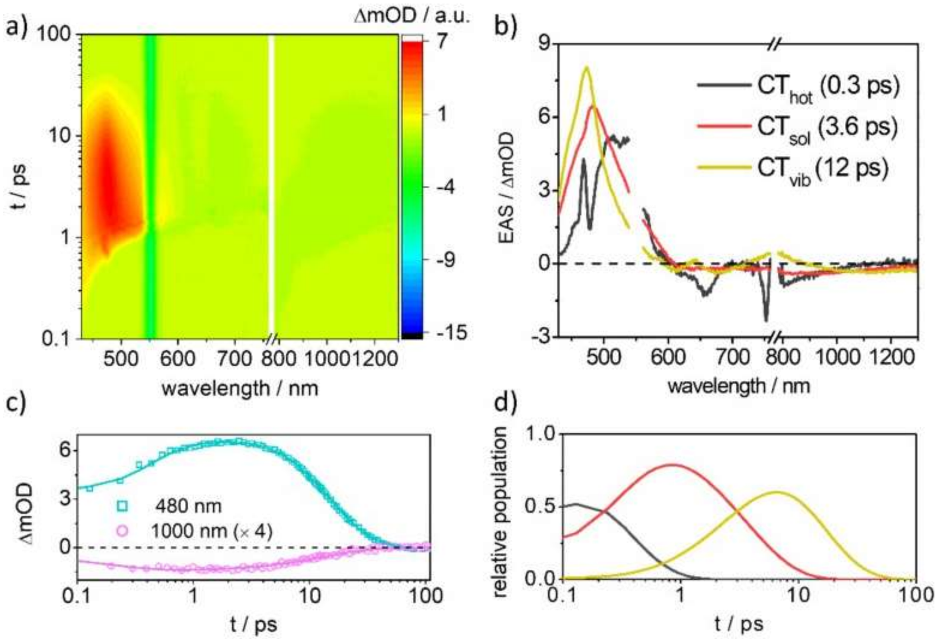

Figure S5.2. a) fs-TAS of **1** (λ_{exc} = 550 nm) with time delays between 0.1 and 100 ps in the visible and near-infrared region in an air-saturated toluene solution at room temperature. b) EAS obtained by a global fit of the fs-TAS data according to a three-species kinetic model in GloTarAn. c) Selected kinetic traces with the corresponding fit. d) Concentration evolution with time related to each species in b).

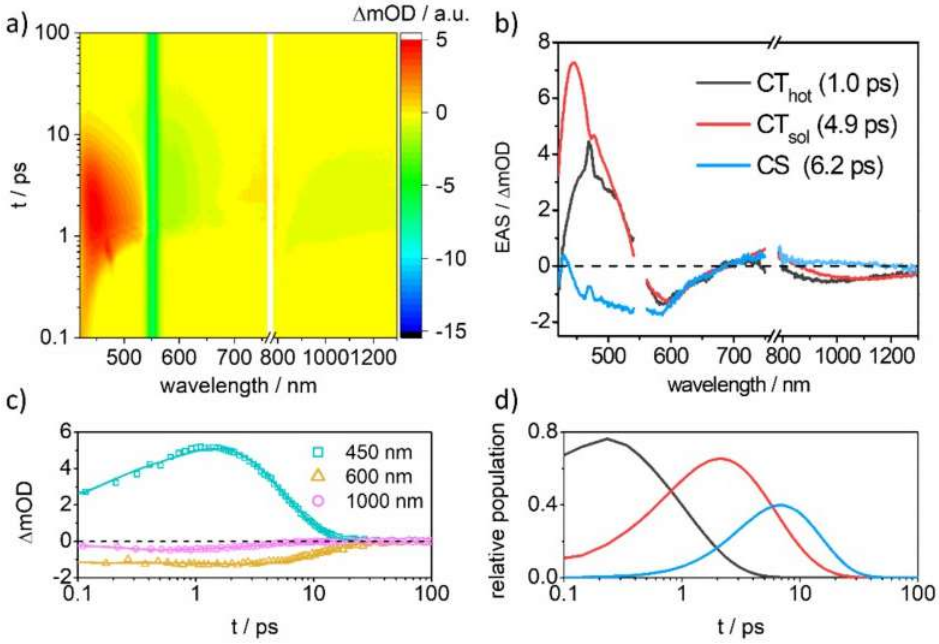

Figure S5.3. a) fs-TAS of **2** (λ_{exc} = 550 nm) with time delays between 0.1 and 100 ps in the visible and near-infrared region in an air-saturated toluene solution at room temperature. b) EAS obtained by a global fit of the fs-TAS data according to a three-species kinetic model in

GloTarAn. c) Selected kinetic traces with the corresponding fit. d) Concentration evolution with time related to each species in b).

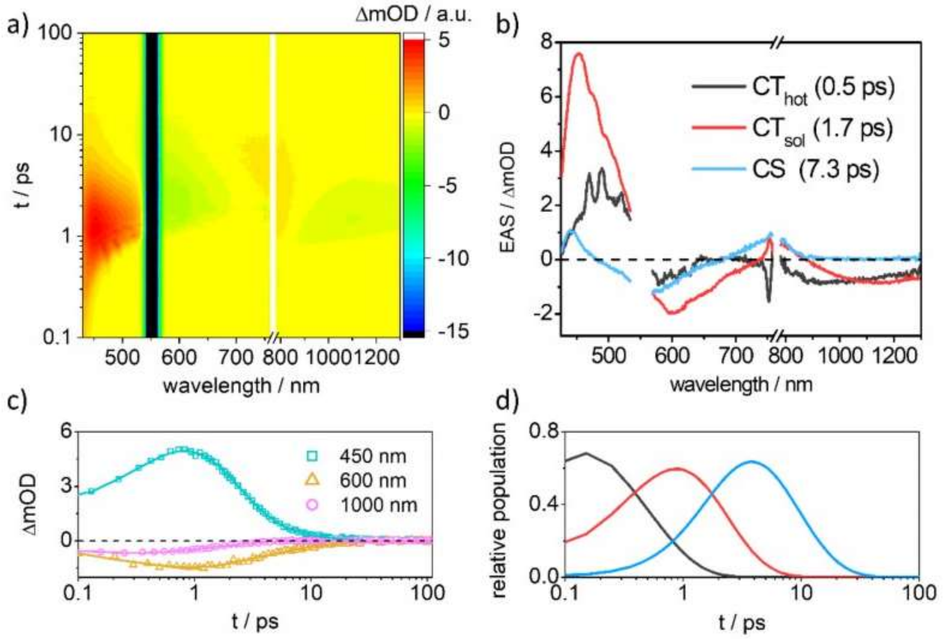

Figure S5.4. a) fs-TAS of **2** (λ_{exc} = 550 nm) with time delays between 0.1 and 100 ps in the visible and near-infrared region in an air-saturated benzonitrile solution at room temperature. b) EAS obtained by a global fit of the fs-TAS data according to a three-species kinetic model in GloTarAn. c) Selected kinetic traces with the corresponding fit. d) Concentration evolution with time related to each species in b).

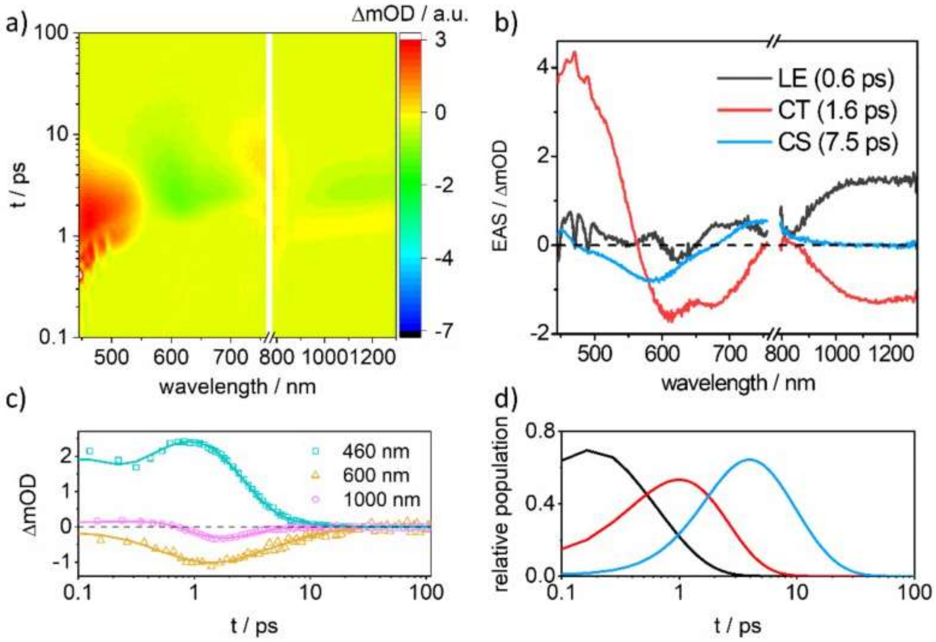

Figure S5.5. a) fs-TAS of **2** (λ_{exc} = 430 nm) with time delays between 0.1 and 100 ps in the visible and near-infrared region in an air-saturated benzonitrile solution at room temperature. b) EAS obtained by a global fit of the fs-TAS data according to a three-species kinetic model in GloTarAn. c) Selected kinetic traces with the corresponding fit. d) Concentration evolution with time related to each species in b).

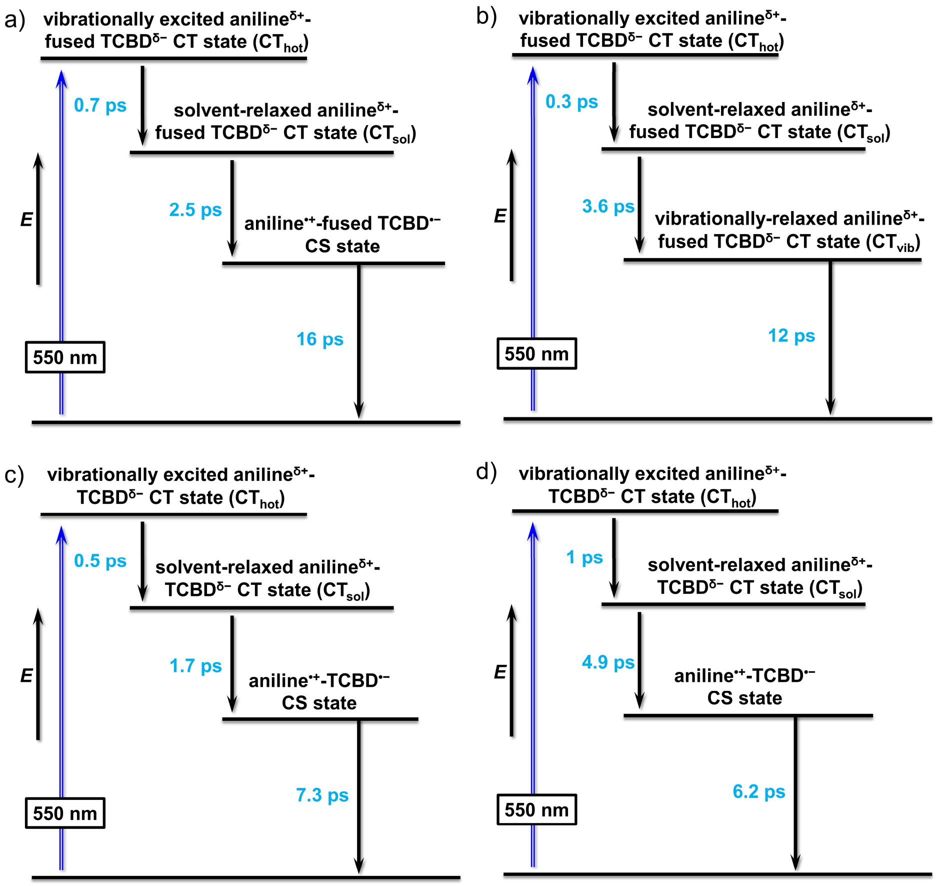

Figure S5.6. Energy level diagrams of a,b) anthryl-fused−TCBD−aniline **1** and c,d) anthryl−TCBD−aniline **2** reflecting the energetic pathways in a,c) benzonitrile and b,d) toluene after excitation at 550 nm. CT = charge transfer; CS = charge separated.

6. References

[1] A. R. Lacy, A. Vogt, C. Boudon, J.-P. Gisselbrecht, W. B. Schweizer, F. Diederich, *Eur. J. Org. Chem.* **2013**, *2013*, 869-879.

[2] M. J. Frisch, G. W. Trucks, H. B. Schlegel, G. E. Scuseria, M. A. Robb, J. R. Cheeseman, G. Scalmani, V. Barone, G. A. Petersson, H. Nakatsuji, X. Li, M. Caricato, A. V. Marenich, J. Bloino, B. G. Janesko, R. Gomperts, B. Mennucci, H. P. Hratchian, J. V. Ortiz, A. F. Izmaylov, J. L. Sonnenberg, Williams, F. Ding, F. Lipparini, F. Egidi, J. Goings, B. Peng, A. Petrone, T. Henderson, D. Ranasinghe, V. G. Zakrzewski, J. Gao, N. Rega, G. Zheng, W. Liang, M. Hada, M. Ehara, K. Toyota, R. Fukuda, J. Hasegawa, M. Ishida, T. Nakajima, Y. Honda, O. Kitao, H. Nakai, T. Vreven, K. Throssell, J. A. Montgomery Jr., J. E. Peralta, F. Ogliaro, M. J. Bearpark, J. J. Heyd, E. N. Brothers, K. N. Kudin, V. N. Staroverov, T. A. Keith, R. Kobayashi, J. Normand, K. Raghavachari, A. P. Rendell, J. C. Burant, S. S. Iyengar, J. Tomasi, M. Cossi, J. M. Millam, M. Klene, C. Adamo, R. Cammi, J. W. Ochterski, R. L. Martin, K. Morokuma, O. Farkas, J. B. Foresman, D. J. Fox, *Gaussian 16 Rev. B.01*, **2017**.

[3] A. D. Becke, *J. Chem. Phys.* **1993**, *98*, 5648-5652.

[4] P. J. Stephens, F. J. Devlin, C. F. Chabalowski, M. J. Frisch, *J. Phys. Chem.* **1994**, *98*, 11623-11627.

[5] T. Yanai, D. P. Tew, N. C. Handy, *Chem. Phys. Lett.* **2004**, *393*, 51-57.

[6] S. Grimme, J. Antony, S. Ehrlich, H. Krieg, *J. Chem. Phys.* **2010**, *132*, 154104.
[7] P. Atkins, J. D. Paula in *Physical Chemistry*, University Press, Oxford pp. 804-805.
[8] T.-I. Ho, A. Elangovan, H.-Y. Hsu, S.-W. Yang, *J. Phys. Chem. B* **2005**, *109*, 8626-8633.
[9] T. Le Bahers, C. Adamo, I. Ciofini, *J. Chem. Theory Comput.* **2011**, *7*, 2498-2506.
